# Single-cell transcriptome analysis of medaka lymphocytes reveals absence of fully mature T cells in the thymus and the T-lineage commitment in the kidney

**DOI:** 10.3389/fimmu.2024.1517467

**Published:** 2025-01-10

**Authors:** Hiyori Sakaguchi, Masaru Matsuda, Norimasa Iwanami

**Affiliations:** Center for Bioscience Research and Education, Utsunomiya University, Utsunomiya, Japan

**Keywords:** medaka, immune system, lymphopoiesis, scRNA-seq, *rag1*

## Abstract

The cellular and molecular mechanisms underlying lymphocyte development are diverse among teleost species. Although recent scRNA-seq analyses of zebrafish hematopoietic cells have advanced our understanding of teleost hematopoiesis, comparative studies using another genetic model, medaka, which is evolutionarily distant among teleosts, is useful for understanding commonality and species-specificity in teleosts. In order to gain insight into how different molecular and cellular mechanisms of lymphocyte development in medaka and zebrafish, we established a *recombination activating gene 1* (*rag1*) mutant medaka, which exhibited defects in V(D)J rearrangement of lymphocyte antigen receptor genes, accordingly lacking mature B and T cells. scRNA-seq analysis of wild type and *rag1* mutant lymphocytes in the thymus and kidney characterized the developing stages of T and B cells, and found that most developed *cd4^+^cd8^–^* and *cd4^–^cd8^+^* single-positive (SP) T-cell populations are absent in the thymus, and identified lymphoid progenitor cells already committed to the T lineage in kidney, implying unique features of medaka lymphocyte development.

## 1 Introduction

Lymphocytes are equipped with highly diverse and specific antigen receptors and play central roles in the adaptive immune systems, which have evolved in vertebrates. In order to secure responses against all kinds of antigens, but at the same time to avoid attacking to self-tissues, lymphocyte development is exquisitely and hierarchically regulated (1, 2). The cellular and molecular mechanisms of mammalian lymphocyte development have been studied in depth, with relevance to such as immune disease patients and model mice (3, 4). V(D)J rearrangement of immunoglobulins and T-cell receptors (TCRs), in which the Recombination-activating gene (RAG) 1 and RAG2 cooperatively play important roles, is indispensable for the development of B cells and T cells, respectively (5–7). Regarding T-cell development in the thymus, the developmental stages of thymocytes are divided by cell surface expression of TCR coreceptors CD4 and CD8 (8), from immature CD4^–^CD8^–^ double-negative (DN), followed by CD4^+^CD8^+^ double-positive (DP), to the most mature CD4^+^CD8^–^ or CD4^–^CD8^+^ single-positive (SP) stages.

Compared to mammals, the mechanisms of the fish immune system, including lymphocyte development, have been much less studied. Teleosts constitute approximately half of the vertebrate species. Recent studies on teleost immune systems using various fish species including zebrafish, which has been established as a model fish for genetics, developmental biology and physiology, have revealed that the principle of adaptive immunity is conserved between teleosts and mammals (9, 10). However, for as many as approximately 425 million years since the divergence of ray-finned fishes including teleosts, and lobe-finned fish including the ancestors of mammals, each group has evolved their adaptive immune systems, resulting in differences in immune cell types and their molecular composition (11).

In addition, recent whole-genome sequencing of teleost species has identified that, even among teleosts, diverse immune systems have been generated. For instance, Atlantic cod lacks major histocompatibility complex (MHC) class II and the entire CD4^+^ T-cell component of adaptive immunity (12, 13). Moreover, a comparative genomics study of deep-sea anglerfish species identified a relationship between the degree of sex parasitism and loss of adaptive immune systems; species with permanent physical attachment even lack functional *rag* genes, resulting in the complete loss of adaptive immune systems (14).

Among such diverse teleost species, zebrafish have been widely used for genetics and bioimaging and have contributed to the understanding of hematopoiesis, lymphopoiesis, and immune functions (10, 15, 16). Zebrafish mutants of orthologs of genes known to be involved in mammalian lymphopoiesis, such as *rag1*, *protein kinase, DNA-activated, catalytic subunit* (*prkdc*), *interleukin 2 receptor, gamma chain* (*il2rg*), exhibit immunodeficiency, indicating the commonality of roles of these genes among vertebrates (17–19).

Although the lack of panels of cell surface antibodies hinders the cellular analysis of lymphocytes, lymphocyte-specific promoter-driven fluorescence transgenic zebrafish have been progressing in understanding the cellular and molecular mechanisms of lymphocyte development and immune functions (20–23). In addition, recent single-cell RNA sequencing (scRNA-seq) analyses of hematopoietic cells from lymphoid organs including the kidney, spleen, and thymus have identified cellular components, their marker genes, and their developing trajectories (24–27). Furthermore, recent single-cell RNA sequencing (scRNA-seq) analyses have identified dynamic changes in cellular components and expression patterns of wild-type and *rag1* mutant gut cells after immune activation (28), as well as in spleen and intestine cells after viral infection (29, 30).

However, as mentioned above, teleosts are evolutionarily diverse; therefore, one model fish cannot represent lymphocyte development in general teleosts. Medaka is a small freshwater fish species that evolutionally diverged from the ancestor of zebrafish as long as 110–160 million years ago, but share similar body size, lifespan, and habitat (31). Medaka has been established as a model organism for studies on genetics, developmental biology, and evolutionary biology (31). The existence of abundant closely related species of medaka that live in a wide range of temperatures and salinities enhances the value of medaka as a model for comparative immunology (32). Recent studies have found that medaka shares fundamental genes involved in hematopoiesis and lymphocyte development with zebrafish and mammals (33–36), indicating its potential as a model for studying the evolution of immune systems. Accordingly, identification of molecular and cellular mechanisms of medaka lymphocyte development and understanding how different mechanisms medaka and zebrafish possess must facilitate comprehension of the evolution of teleost immune systems.

Here, to uncover the molecular and cellular mechanisms of medaka lymphocyte development, we established an immunodeficient *rag1* mutant medaka. The *rag1* mutant exhibited defects in the V(D)J rearrangement of *immunoglobulin* and *t-cell receptor* genes, and accordingly lacked mature B and T cells. scRNA-seq analysis of wild-type and *rag1* mutant lymphocytes in the kidney and thymus identified *rag1*-independent and -dependent developing lymphocytes. We characterized the developmental stages of B and T cells, and found that there was neither a *cd4^+^cd8^–^* nor *cd4^–^cd8^+^* single-positive (SP) population in the thymus and identified lymphoid progenitor cells expressing novel family genes encoding immunoglobulin domain-containing cell surface proteins already committed to the T lineage in the kidney. These mechanisms are unique features of medaka lymphocyte development and are not shared by zebrafish.

## 2 Materials and methods

### 2.1 Fish lines

The medaka (*Oryzias latipes*) lines were obtained from the National Bioresource Project (NBRP) Medaka and maintained at Utsunomiya University: standard line OK-cab (strain ID: MT830) and cab-Tg (*rag1:egfp*), a transgenic medaka expressing *egfp* under the control of an immature lymphocyte-specific *rag1* promoter (strain ID: TG848) (34). Fish were maintained in temperature-controlled tanks (26°C) with a water circulation system under a 14 h light/10 h dark cycle. This study was conducted in accordance with the ethical guidelines of the Utsunomiya University Animal Experimentation Committee and the experimental protocols were approved by the committee (approval no. A23-0011). The developmental stages of medaka were designated as previously described (37).

### 2.2 Genome editing

The Alt-R CRISPR-Cas9 crRNA targeting exon 4 of *rag1* (ENSORLG00000011969.3), Alt-R CRISPR-Cas9 tracrRNA, and Cas9 protein were purchased from Integrated DNA Technologies (IDT; Singapore). The sgRNA sequences are listed in **Supplementary Table S1**. RNA–protein complexes were prepared according to the manufacturer’s instructions. Glass capillaries with 1.0 mm outer diameter were pulled (temperature 62°C, force: two light and two heavy weights provided by the manufacturer) using a micropipette puller PC-10 (Narishige Instruments, Tokyo, Japan). The capillary was filled with crRNA (0.75 mM), tracrRNA (1.5 mM), and 0.25 mg/mL Cas9 protein. Then, 1–2 nL of the mixture was injected into 1-cell Cab embryos using FemtoJet 4i (Eppendorf, Hamburg, Germany). G0 fish were crossed with wild-type (WT) Cab fish to establish the *rag1* mutant strains. *rag1^del8^* was chosen for the phenotypic analysis. The primers used for genotyping are listed in **Supplementary Table S1**. The mutant line was deposited in NBRP medaka as rag1 (del8); MT1583.

### 2.3 Transgenesis

The 4.7kb of the medaka *lck* promoter, upstream of the ATG initiation codon situated in exon 2, was amplified from the genomic DNA of the Hd-rR strain. *egfp* and SV40 polyA were amplified from pEGFP-1 plasmid (Takara Clontech). Primer sequences are listed in **Supplementary Table S1**. Amplicons of the *lck* promoter and *egfp*+SV40 polyA were cloned into the linearized pUC19 plasmid using In-Fusion HD (Takara Clontech). Accordingly, the *lck:egfp* construct, including the medaka *lck* promoter, *egfp*, and SV40 polyA, was generated. 25 ng/µL *lck:egfp* construct was injected into fertilized eggs of the cab strain for transgenesis using FemtoJet 4i (Eppendorf, Hamburg, Germany). Embryos around the day of hatching were observed under a fluorescence microscope and those exhibiting EGFP signals in the pharyngeal region as was previously reported (38) were further used to establish *lck:egfp* transgenic lines. Two stable transgenic lines were generated, one of which was used for further analysis. The transgenic line was deposited in NBRP Medaka as cab-Tg (lck-egfp); TG1582.

### 2.4 Imaging of medaka specimens

Larvae and adult medaka were anesthetized with 0.1% (v/v) 2-phenoxyethanol and immobilized in 3% methylcellulose. Images were captured using a DFC450c digital camera (Leica, Houston, TX, USA) under an M205FA fluorescence stereo microscope (Leica, Houston, TX, USA) and acquired using Leica Application Suite X (LASX) v1.1.0 (Leica, Houston, TX, USA). The area and fluorescence intensity of the images were measured using the ImageJ software (National Institutes of Health, MD).

### 2.5 Histological analysis

Whole bodies of the adults were fixed in 4% Paraformaldehyde/PBS overnight at 4°C. Paraffin sectioning was performed as previously described (39). Transverse sections (5 µm thick) were stained with hematoxylin and eosin. Images were captured using a DP73 digital camera (Olympus, Tokyo, Japan) under a BX60 microscope (Olympus, Tokyo, Japan).

### 2.6 Reverse transcription-PCR

RNA was extracted using a NucleoSpin RNA kit (Macherey-Nagel, Düren, Germany) with on-column DNase treatment. RNA was reverse-transcribed using a QuantiTect Reverse Transcription Kit (Qiagen). To detect *immunoglobulin mu (igm)* and *t-cell receptor beta chain (tcrb)* cDNA after VDJ rearrangement, nested PCR was performed using primer sets targeting the variable (V) and constant (C) regions. Two V regions that were highly expressed in wild-type kidney were chosen for RT-PCR analysis (data not shown). Amplification was performed in a thermal cycler using the following program: 1 min at 95°C; cycles of 10 s at 95°C, 30 s at 59°C, 30 s at 72°C; and 5 min at 72°C. The cycle numbers were as follows: *actb* (*actin beta*), 25 cycles; *igm* (igV_H_-Cm) and *tcrb* (tcrVb-Cb2), 30 cycles plus nested 30 cycles. The primers used for the RT-PCR are listed in **Supplementary Table S1**.

Quantitative PCR (qPCR) was performed using the THUNDERBIRD SYBR qPCR Mix (Toyobo, Osaka, Japan) and LightCycler 96 (Roche, Basel, Switzerland). Primers used for qPCR are listed in **Supplementary Table S1**.

### 2.7 Flow cytometry

Whole kidney marrow (WKM) cells and thymocytes were prepared as previously described (36). Cells were obtained by pipetting the organs into 1 mL ice-cold 1% fetal bovine serum (FBS) in 0.9× PBS, washed with 1% FBS in 0.9× PBS by centrifugation, and then filtered using a 40µm-stainless mesh. Analytical flow cytometry (FCM) was performed on a FACSLyric flow cytometer (BD Biosciences). Populations of lymphocytes, myelomonocytes, and precursors in WKM cells in light scatter profiles were defined using previous information in zebrafish and medaka (40, 41). Data analysis was performed using the Flowjo10.6.2 (BD Biosciences).

FACS Aria III (BD Biosciences) was used for cell sorting. The cell populations in the light scatter profiles of WKM cells and thymocytes were defined according to previous studies (34, 40, 41).

### 2.8 scRNA-seq

The single-cell RNA-seq library was constructed using the Chromium Controller and Chromium Next GEM Single Cell 3′Reagent Kits v3.1 (10x Genomics) following the standard manufacturer’s protocols. The FACS-sorted single-cell suspension was immediately loaded onto 10x Chromium iX to recover 10000 cells, followed by library construction. The library was sequenced using the Novaseq6000 system (Illumina), according to the manufacturer’s instructions. Sequencing was performed using a 28/90bp paired-end configuration. The obtained dataset was processed with Cell Ranger (v7.2.0). Oryzias_latipes.ASM223467v1.dna.toplevel.fa.gz (Ensembl) and olatipes-HdRr-ensembl-peaks2utr.v1.gtf.gz (https://figshare.com/articles/dataset/Medaka_Gene_Model_version_1/24080463?file=42403659) were used as the reference genome and annotation files, respectively.

### 2.9 Cell characterization and identification of differentially expressed genes

Loupe Browser v7.0.1 and v8.1.2 were used to characterize cell types. Barcodes with total Unique Molecular Identifier (UMI) counts of 2048–32768 (2^11^–2^15^) were used for this analysis. Cell characterization was based on gene expression profiles in t-distributed Stochastic Neighbor Embedding (t-SNE) plots. The proportion of clusters per sample was normalized, and principal component analysis (PCA) was performed using Python library scikit-learn (42). Differentially expressed genes were obtained by “compared to entire dataset” mode based on each cluster. The p-values calculated using the differential expression analysis feature were adjusted for multiple comparisons using the Benjamini–Hochberg correction. Python package Scanpy v1.9.3 (43) (https://genomebiology.biomedcentral.com/articles/10.1186/s13059-017-1382-0) was used to generate heatmaps. Genes used for cluster characterization were chosen based on expression profiles in zebrafish and mice (22, 25–27, 44–50) (**Supplementary Table S1**).

*ighm, sid1, trac*, and *trdc* were not annotated in medaka Ensembl ASM223467v1. Reads mapped to the target region and their cell barcodes were obtained from the bam files generated by Cell Ranger using SAMtools (ver. 1.10). Target areas were defined as *ighm* (8:14941689-14941969) (34) *sid1* (4:24323750-24324064), *trac* (17:12460290-12460457) (34) and *trdc* (17:12510953-12511133) (51). *sid1* was retrieved from the genome database of Ensembl ASM223467v1 through TBLASTN, using the amino acid sequence of zebrafish *sid1* (ENSDARG00000095540) as a query. Zebrafish and medaka *sid1* are commonly located next to *si:ch211-1a19.3*. The frequency of each cell barcode in the generated bam file was counted using the awk command, and a csv file containing the cell barcodes and their frequencies was imported into Loupe Browser’s cluster mode for visualization on a t-SNE plot.

### 2.10 Gene ontology analysis

GO enrichment analysis was performed for 109 upregulated genes and 79 downregulated genes in the DP T (1) cluster against the DP T (2) cluster using Metascape v3.5.20240901 (https://metascape.org/gp/index.html#/main/step1) (52). Analysis was performed using annotation datasets of complete GO biological processes and a reference list of human genes. Twelve upregulated and 20 downregulated genes that were uncharacterized or lacked human orthologs were excluded from the analysis.

### 2.11 Data deposition

The datasets generated in the current study are available from the DNA Data Bank of Japan (DDBJ) database (accession numbers DRA013138 and DRA017334).

## 3 Results

### 3.1 Establishment of *rag1* mutant medaka lacking mature lymphocytes

**Figure 1:**
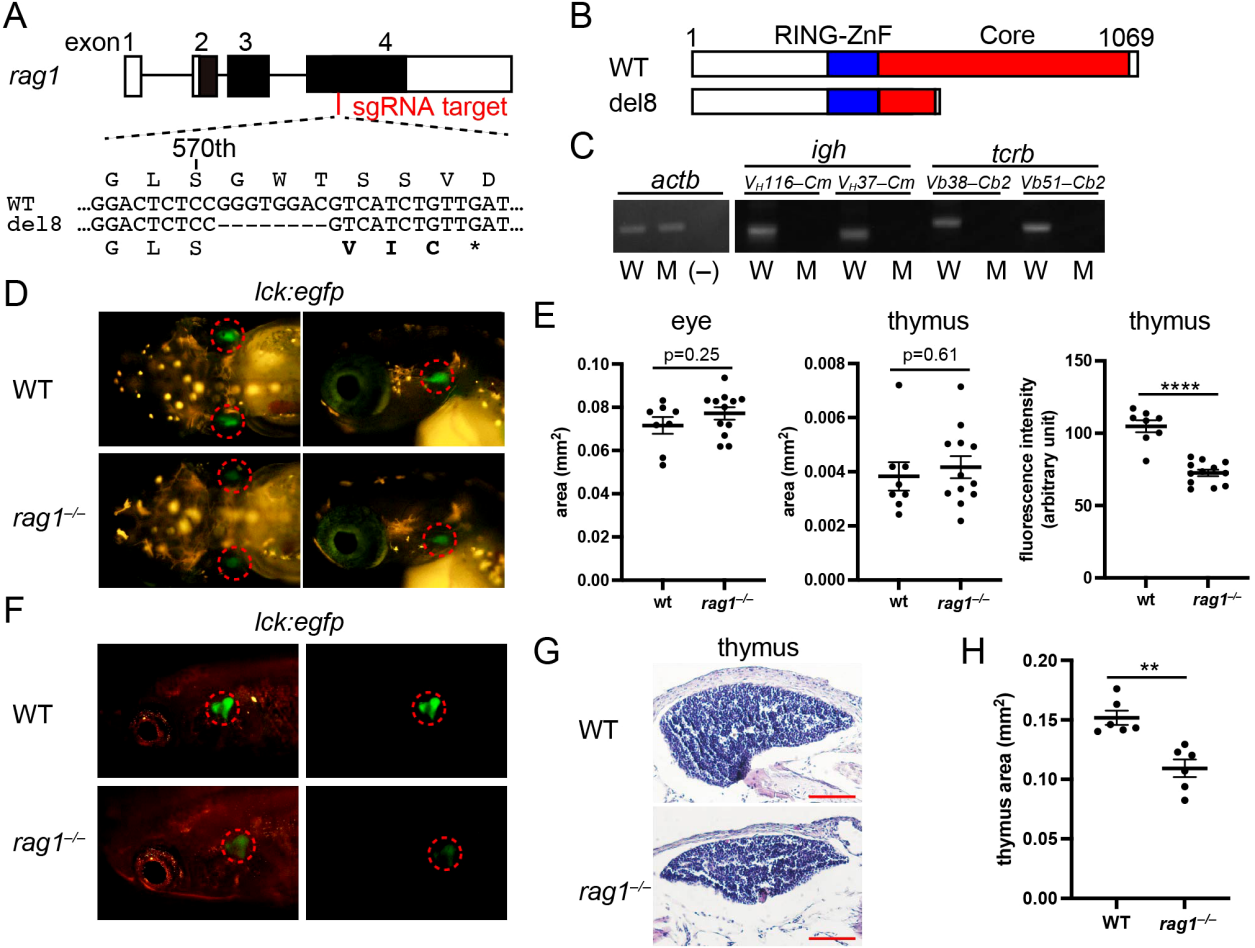
Generation of *rag1* mutation by genome editing. **(A)** Exon/intron structure of the medaka *rag1* gene (top). Closed boxes indicate coding exons. Open boxes indicate the untranslated exons. sgRNA target for genome editing is indicated in red font. Nucleotide and deduced amino acid sequences of the wild-type and 8 bp deletion mutant in exon 4 (bottom). Bold letters indicate unrelated amino acids. * indicates the stop codon. **(B)** Domain structures of the wild-type and del8 mutant Rag1 proteins. RING domain and zinc-finger domain (RING-ZnF) and Core region (Core) are indicated in blue and red, respectively. **(C)** Reverse transcription PCR (RT-PCR) of whole kidney cells of the indicated genotypes, using the indicated primers to detect VDJ-recombined *igm* and *tcrb*. *actb* expression was used as the standard. (−), water control; W, wild type; M, *rag1^–/–^*. **(D)** *lck:*EGFP expression in larval thymocytes at 10 days post-fertilization (dpf). Dorsal (left) and lateral (right) views of the indicated genotypes. Dotted circles indicate the thymus. **(E)** Plots of the area of the eye in the corresponding visible-light images (left) and EGFP^+^ thymus (middle), and the fluorescence intensity of EGFP in the thymus (right), measured using dorsal views of **(E)** Data represent the mean ± SEM; statistical significance was determined using an unpaired two-tailed t-test. ****p < 0.0001. **(F)** *lck:*EGFP expression in adult thymocytes at 2 months post-fertilization (mpf). Lateral views of the indicated genotypes with (left) and without (right) visible-light transmission. Dotted circles indicate the thymus. Note the less intense EGFP signals of thymocytes in *rag1* mutants. **(G)** Transverse sections (5 µm thick) of the adult thymus were stained with hematoxylin and eosin. Scale bars: 100 µm. **(H)** Plots of the area of the largest portions of thymi in the indicated genotypes. Data represent mean ± SEM; statistical significance was determined using an unpaired two-tailed t-test. **p < 0.01.

To establish an immunodeficient genetic model that lacks mature lymphocytes and use it for the analysis of cellular mechanisms of lymphocyte development, *rag1* mutant medaka (*rag1^–/–^*) were generated by genome editing using the CRISPR/Cas9 system, targeting exon 4 of *the rag1* gene (**Figure 1A**). A mutant line with an 8 bp deletion in *rag1* was established, which was predicted to cause a frameshift and encode 570 appropriate amino acids, followed by three unrelated amino acids and a stop codon (**Figure 1A**). The deduced protein lacked most of the core region including the central domain, which recognizes the recombination signal sequence heptamer and RAG2 (53, 54) (**Figure 1B**), and was considered a null allele. The homozygous form of this frameshift mutant did not exhibit gross developmental defects.

First, we investigated the transcripts of rearranged lymphocyte antigen receptors in the kidney, which is equivalent to mammalian bone marrow as a site of hematopoiesis. Reverse transcription PCR (RT-PCR) analysis did not detect VDJ-recombined *igm* transcripts in B cells or *tcrb* transcripts in T cells in *the rag1* mutant (**Figure 1C**; **Supplementary Figure S1**), as expected. Crossing the mutant with *rag1:egfp* transgenic medaka, which expresses EGFP under the control of the immature lymphocyte-specific *rag1* promoter (34), revealed a reduction in EGFP-expressing cells in the larval thymus (**Supplementary Figure S2A**). Crossing the mutant with *lck:egfp* transgenic medaka (**Supplementary Information**, **Supplementary Figure S3**), which expresses EGFP under the control of the immature T and NK cell-specific *lck* promoter, revealed a comparative area of EGFP-expressing cells in the larval thymus (**Figures 1D, E**). However, the fluorescence intensity of the thymus area in *the rag1* mutant was significantly reduced compared to that in the wild-type (**Figure 1E**), suggesting a lower *lck*:EGFP^+^ cell density in the *rag1* mutant. Adult *rag1* mutant of *rag1:egfp* and *lck:egfp* backgrounds exhibited a reduction in EGFP signals in the thymus (**Supplementary Figure S2B**; **Figure 1F**). Furthermore, reduction of thymus size in *the rag1* mutant was confirmed by tissue sections (**Figures 1G, H**). These results suggest that the *rag1* mutant has defects in T-cell development.

**Figure 2:**
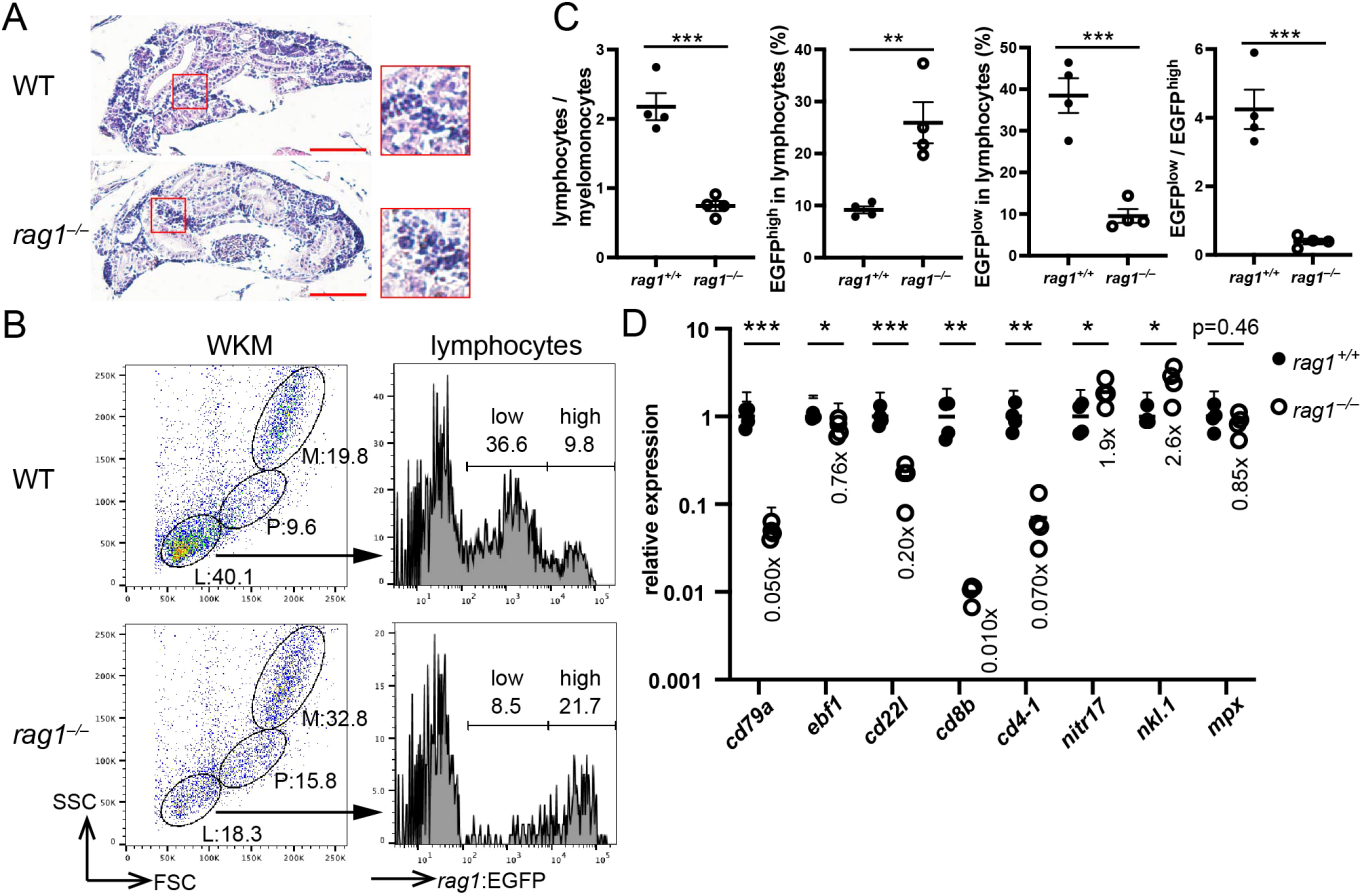
*rag1* mutation affects B- and T- cell development. **(A)** Transverse sections (5 µm thick) of adult kidney of the indicated genotypes were stained with hematoxylin and eosin. Scale bars: 100 µm. Magnified images of the areas of hematopoietic cells (red squares) are also shown. Note the comparable cellularity of the kidney in *the rag1* mutant. **(B)** Flow cytometric profiles of adult whole kidney marrow (WKM) cells of indicated genotype with *rag1:egfp* transgene. Light scatter profiles (left). Circles indicate lymphocyte (L), precursor (P), and myelomonocyte (M) populations. EGFP signal levels in lymphocyte populations (right). Numbers indicate proportions (%). FSC, forward scatter; SSC, side scatter. **(C)** Plots of lymphocytes/myelomonocytes ratio of WKM cells (left), percentage of EGFP^high^ cells in lymphocytes (2nd from left), percentage of EGFP^low^ cells in lymphocytes (2nd from right), and EGFP^low^/EGFP^high^ ratio (right). Data represent mean ± SEM; statistical significance was determined using an unpaired two-tailed t-test. **p < 0.01; ***p<0.001. **(D)** Quantitative PCR (qPCR) analysis of the kidney cells of the indicated genotypes (n=4). The average expression level in *rag1^+/+^* is normalized to 1. Data represent the mean ± standard error of the mean (SEM), and statistical significance was determined using an unpaired two-tailed t-test. *p < 0.05; **p < 0.01; ***p<0.001. The average expression level of each gene in *the rag1* mutant is also shown. The results represent one of the two independent experiments with similar results.

Next, B-cell development in the kidney of *the rag1* mutant was assessed. Kidney sections from wild-type and *rag1* mutants showed a comparable appearance of hematoxylin-rich blood cells (**Figure 2A**). Fluorescence-activated cell sorting (FACS) analysis of WKM cells was carried out for *the rag1* mutant of *the rag1:egfp* background. Light scatter profiles revealed that the proportion of lymphocytes, which are mostly developing B cells (explained later in detail), against myelomonocytes was severely reduced in *the rag1* mutant (**Figures 2B, C**). EGFP expression levels in the lymphocyte population were different between the wild type and *rag1* mutant; whereas *the rag1*:EGFP^high^ population corresponding to relatively immature B cells was increased in *the rag1* mutant, *rag1*:EGFP^low^ population corresponding to relatively mature B cells was decreased in *the rag1* mutant (**Figures 2B, C**, explained later in detail). In qPCR analysis of adult WKM cells, the expression of pan B-cell marker *cd79a* was significantly reduced. Contrary to a mild decrease in the expression of the early B-cell marker *ebf1*, expression of the relatively mature B-cell marker *cd22l* was severely reduced in *the rag1* mutant, suggesting arrested B cell-development in the kidney. In addition, the expression of the T-cell marker *cd8b* was significantly decreased in *rag1* mutants (**Figure 2D**). In contrast, the expression of the natural killer cell markers *nitr17* and *nkl.1* was mildly increased in *rag1* mutants (**Figure 2D**). Among the other blood populations, the expression of the neutrophil marker *mpx* was comparable (**Figure 2D**). These results suggest that *rag1* mutations affect T- and B- cell development.

### 3.2 Identification of stages of T-cell development in the thymus

**Figure 3:**
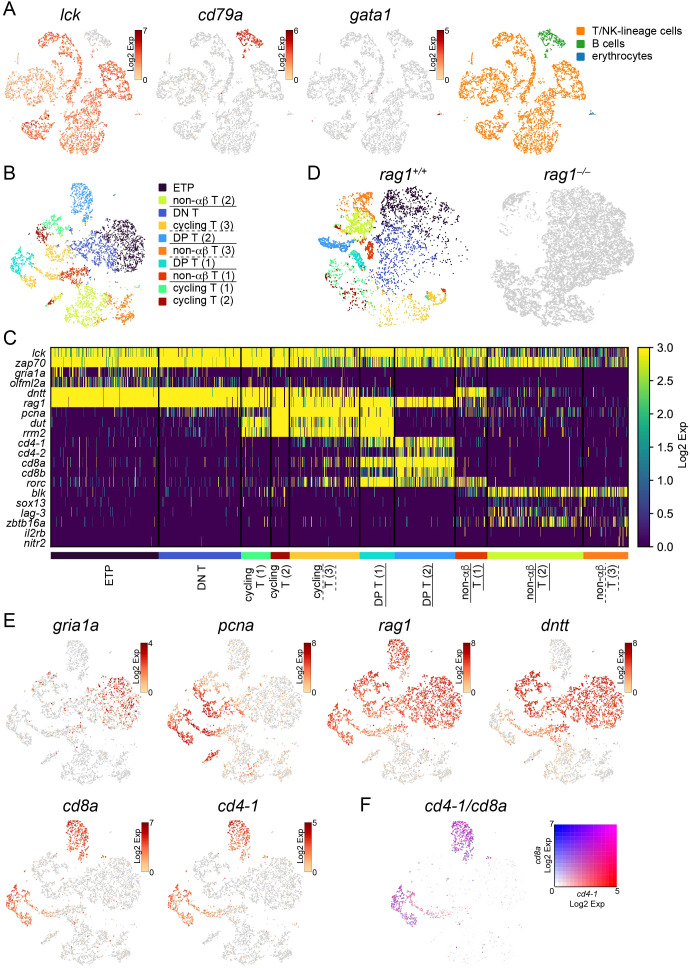
Categorization of developing T cells in the thymus. **(A)** t-SNE visualization of 6192 lymphocytes from the wild-type thymus. The expression levels of the indicated genes (left) and categorization of 12 clusters based on the expression of these genes (right) are shown. **(B)** t-SNE visualization of 5730 T/NK-lineage cells from wild-type thymus. Clusters with annotations were also presented. *rag1*-dependent clusters which are absent in the *rag1* mutant in **(D)** are underlined. Clusters that are *rag1*-dependent in part are underlined with dashed lines. ETP, early T-cell progenitors; DP, double-positive. **(C)** Heatmap of marker gene expression in each T/NK-lineage cell cluster in wild-type. *rag1*-dependent clusters which are absent in the *rag1* mutant in **(D)** are underlined. Clusters that are *rag1*-dependent in part are underlined with dashed lines. **(D)** t-SNE visualization of T/NK-lineage cells from wild-type (left) and *rag1* mutant (right) thymus. A total of 5730 wild type and 12661 mutant cells are shown. Wild-type cells are colored based on the clusters in **(B)**. Mutant cells are shown in gray. **(E)** t-SNE visualization of T/NK-lineage cells from wild-type thymus. Expression levels of indicated genes are shown. **(F)** t-SNE visualization of T/NK-lineage cells from the wild-type thymus. The merged expression levels of *cd4-1* and *cd8a* are shown.

To analyze the cellular and molecular mechanisms of T-cell development, thymocytes from two wild-type and two *rag1* mutants at five months post-fertilization (mpf) were harvested separately. Cell sorting using light scatters was performed to obtain lymphocytes for single-cell transcriptome profiling (**Supplementary Figure S4**). It was confirmed that the lymphocyte gate used for cell sorting covered the *lck*:EGFP^+^ cells (**Supplementary Figure S4**). 6192 cells out of the 7390 wild-type cells, and 13239 cells out of 14528 *rag1* mutant cells had appropriate UMI numbers and were accordingly used for clustering.

t-SNE visualization separated wild-type lymphocytes into 12 clusters, including 11 *lck*-expressing T/NK-lineage clusters and one *cd79a*-expressing B-cell cluster (**Figure 3A**). *gata1*-expressing erythrocytes, which seem to be contaminated by forward scatter (FSC)^low^ erythrocytes during cell sorting, were not many enough to constitute an independent cluster (**Figure 3A**). Excluding this B cluster and erythrocytes from wild-type and the corresponding *rag1* mutant cells, PCA analysis of 5730 cells from two wild-type and totally 12661 cells from *rag1* mutant cells based on the proportion of cells in each cluster was performed, confirming genotype-dependent separation (**Supplementary Figure S5A**).

Next, 5730 wild-type cells in T/NK-lineage clusters were separated into 10 clusters and annotated according to subsequent analyses of gene expression patterns and *rag1*-dependency as described below (**Figure 3B**). A heat map of gene expression levels was constructed using each cluster-specific representative genes (p<0.05) and genes known to be involved in T-cell development in mammals and zebrafish (**Figure 3C**; **Supplementary Tables S2**, **S3**). *lck* and *zap70* were constitutively expressed in all T/NK-lineage clusters (**Figure 3C**). Indication of wild-type T/NK-lineage clusters on t-SNE visualization of T/NK-lineage cells of both wild-type (5730 cells) and *rag1* mutant (12661 cells) identified *rag1*-independent clusters (shared by wild-type and *rag1* mutant) of early T-cell development and *rag1*-dependent clusters (absent in *rag1* mutant) of late T-cell development requiring V(D)J recombination of *tcr* genes (**Figure 3D**). In contrast, re-clustering of T/NK cells from a mixture of wild-type and *rag1* mutants did not generate any *rag1* mutant-specific clusters (data not shown).

Cells of all five *rag1*-independent early clusters [Early T-cell progenitors (ETP), DN T, and cycling T (1–3)] were highly accumulated in *the rag1* mutant (**Figure 3D**), suggesting developmental arrest at these stages. These clusters expressed *rag1* and *dntt* (**Figures 3C, E**). In addition, two *rag1*-dependent clusters [DP T (1–2)] expressed *rag1*, *cd4*, and *cd8*, but not *dntt* (**Figures 3C–E**). Genes labeling proliferating cells (*pcna*, *dut*, *rrm2*) were commonly expressed in four clusters [cycling T (1–3), DP T (1)] (**Figures 3C, E**), suggesting cell proliferation before *tcra* or *tcrb* gene rearrangement. These results imply that cycling T (1–3) clusters are in the course of proliferation before *tcrb* gene rearrangement, and DP T (1–2) clusters expressing both *cd4* and *cd8* are in the more mature stage of T-cell development with ongoing or completed *tcra* gene rearrangement. These *cd4^+^cd8^+^* double-positive (DP) T clusters commonly expressed *rorc* (**Figure 3C**).

The remaining *rag1*-independent *cd4^–^cd8^–^* double-negative (DN) clusters without proliferative gene expression (ETP and DN T) were assumed to be the most immature T cells. Although ETPs and genes specifically expressed in ETPs have been identified in zebrafish (27), these genes including *cebpa*, *csf1rb*, and *fli1* were not expressed in these clusters in medaka (data not shown), suggesting that the timing of ETP immigration or gene expression patterns of ETPs is not conserved between zebrafish and medaka. Instead, the ETP cluster, not the DN T cluster, highly expressed some genes including *gria1a* and *olfml2a* (**Figures 3C, E**), suggesting that this ETP cluster contains recent thymic immigrants already expressing *rag1*.

### 3.3 Absence of the most developed SP T-cell populations in the wild-type thymus

In the t-SNE profile of wild-type T/NK-lineage cells, two clusters expressing *cd8a* and *cd4-1* [DP T (1–2)] were located separately (**Figures 3B, E**). *cd8b*, whose gene product is assumed to form a heterodimer with the gene product of *cd8a*, and *cd4-2*, a teleost-specific paralogous gene of *cd4-1*, are widely expressed in the DP T (2) cluster. In contrast, *cd8b*- and *cd4-2*-expressing cells in the DP T (1) cluster were limited (**Figure 3C**; **Supplementary Figure S6A**). Notably, neither *cd4-1^+^cd8a^–^* nor *cd4-1^–^cd8a^+^* SP clusters were detected (**Figures 3C, E**; **Supplementary Figure S6A**). Looking at the expression levels at the individual cell level, these genes were expressed in cells in the DP T clusters in an overlapping manner (**Figure 3F**).

**Figure 4:**
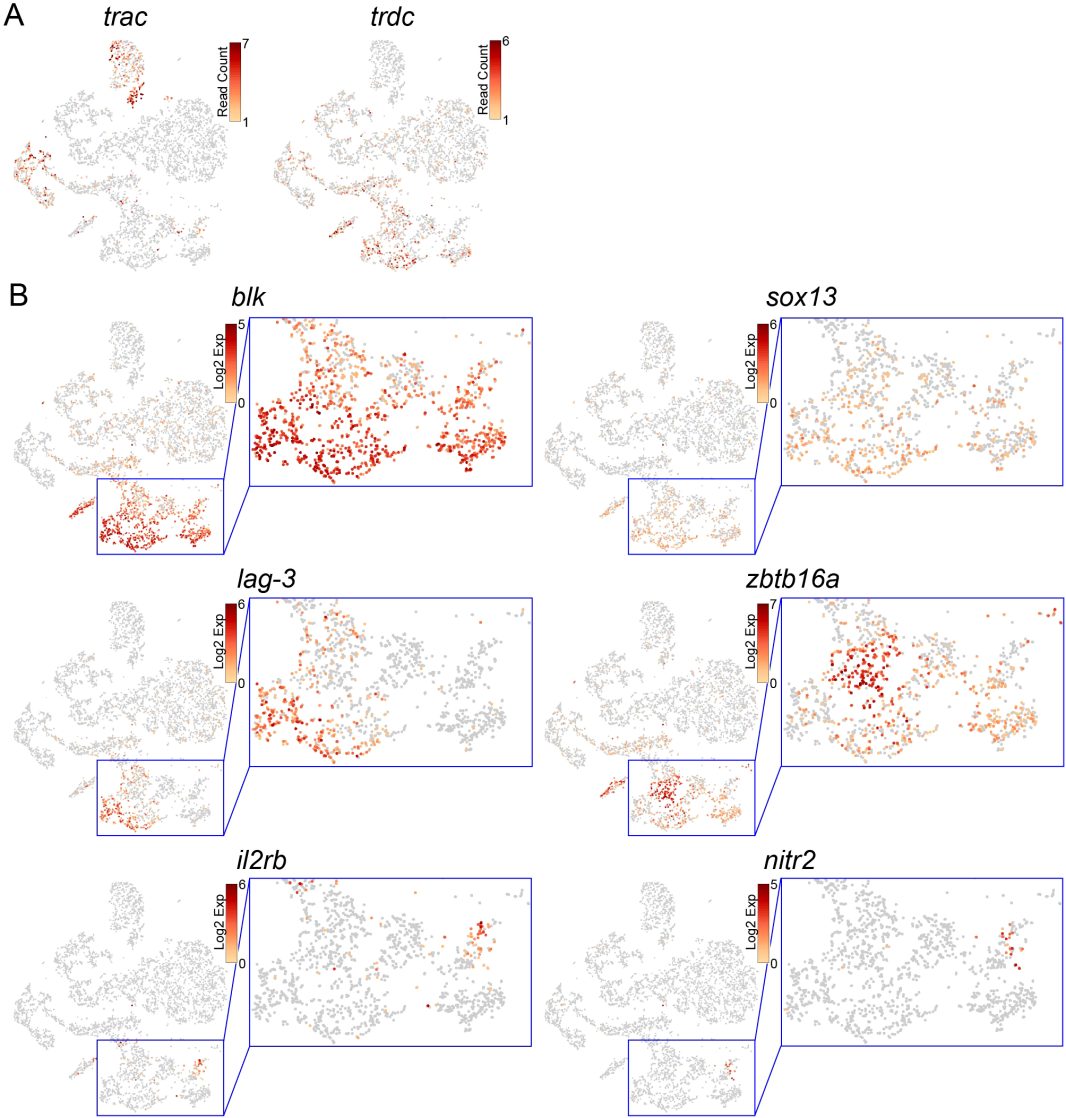
Identification of γδ T, NKT, and NK cells in the thymus. **(A, B)** t-SNE visualization of T/NK-lineage cells from the wild-type thymus. Expression levels of indicated genes are shown. Magnified images of the blue square areas in **(B)** are also shown.

To characterize the two DP T clusters, gene expression profiles were compared between them, and 109 and 79 genes were identified to be highly expressed in DP T (1) and DP T (2), respectively (p<0.05, log_2_fc>2) (**Supplementary Table S4**). GO analysis showed that genes related to pathways for cell division including “DNA replication-dependent chromatin assembly” (*rbbp4*, *chaf1a*, and *asf1b*) and “spindle elongation” (*incenp*, *kif11*, *prc1*, *aurkb*, *kif23*, and *cdca8*) were significantly enriched in DP T (1) cluster (**Supplementary Figures S6B, D, F**), suggesting that cells in DP T (1) cluster are more actively proliferating. On the other hand, genes related to pathways for lymphopoiesis including “T cell differentiation” and “T cell activation” (*cd4-1*, *cd4-2*, *cd8a*, *cd8b*, *egr1*, *itk*, *ptger4*, *tespa1*, and *ccr9*) were significantly enriched in DP T (2) cluster (**Supplementary Figures S6C, E, F**), suggesting that cells in DP T (2) cluster are more mature T cells. Notably, *tespa1*, which is required for positive selection in mammals (55), was enriched in the DP T (2) cluster. Both DP clusters express *trac* (**Figure 4A**), confirming that they are at the most mature stage of αβ T-cell development.

### 3.4 Identification of γδT, NKT, and NK cells in the thymus

In contrast to *trac* expression in the two DP T clusters, *trdc* was expressed in a part of the three clusters [non-αβ T (1–3)] of the wild-type T/NK-lineage cells (**Figure 4A**). Similarly, *sox13* and *blk*, which marked thymic γδ T cells in zebrafish (27), were also expressed in some cells in these three clusters (**Figures 4B**, **3C**), suggesting that these clusters include γδ T-lineage cells. The expression of *lymphocyte activation gene-3* (*lag-3*) encoding a CD4-related inhibitory receptor (56) was overlapped with the area of γδ T cells (**Figures 4B**, **3C**). In-depth observation of these clusters showed that they also contained cells highly expressing *zbtb16a*, which encodes the natural killer T (NKT)-lineage-specific transcription factor Plzf (57, 58) (**Figures 4B**, **3C**). *zbtb16a* expression did not merge with the area of γδ T cells. In addition, we found a *rag1*-independent cell mass expressing the natural killer (NK)-cell markers *il2rb* and *nitr2* (25, 26, 59) (**Figures 4B**, **3C**). These results suggest that, although there is no clear cluster distinction, the three non-αβ T clusters contain γδ T, NKT, and NK cells.

### 3.5 Identification of stages of B-cell development in the kidney

**Figure 5:**
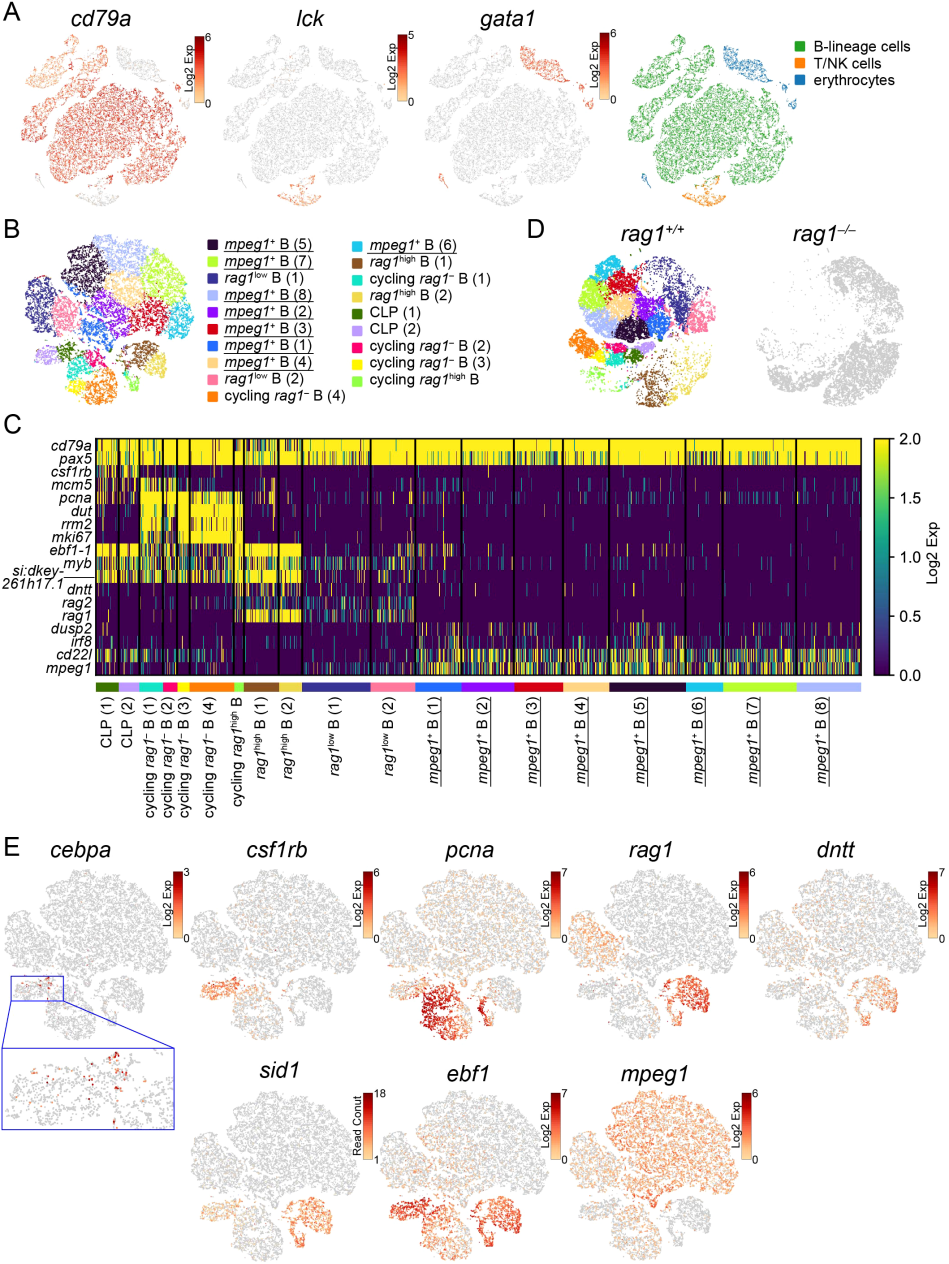
Categorization of developing B cells in the kidney. **(A)** t-SNE visualization of 22929 lymphocytes from the wild-type kidney marrow. The expression levels of the indicated genes (left) and categorization of 24 clusters based on the expression of these genes (right) are shown. CLP, common lymphoid progenitors. **(B)** t-SNE visualization of 19804 B-lineage cells from the wild-type kidney marrow. Clusters with annotations are also presented. *rag1*-dependent clusters which are absent in *rag1* mutant in **(D)** are underlined. **(C)** Heatmap of marker gene expression in each B-lineage cell cluster in the wild-type. *rag1*-dependent clusters which are absent in *rag1* mutant in **(D)** are underlined. **(D)** t-SNE visualization of B-lineage cells from wild-type (left) and *rag1* mutant (right) kidney marrow. 19804 wild type and 12989 mutant cells are shown. Wild-type cells are colored based on the clusters in **(B)**. Mutant cells are shown in gray. **(E)** t-SNE visualization of B-lineage cells from the wild-type kidney marrow. Expression levels of indicated genes are shown. A magnified image of the blue square area is also shown.

To assess the cellular and molecular basis of B-cell development, WKM cells from the three wild-type and two *rag1* mutants at 2 mpf were harvested separately. Cell sorting using light scatters was carried out to obtain lymphocytes for single-cell transcriptome profiling using Cell Ranger (10x Genomics) (**Supplementary Figure S4**). 22929 cells out of 25103 wild-type cells and 15472 cells out of 17604 *rag1* mutant cells had appropriate UMI numbers, and accordingly were used for clustering.

t-SNE visualization separated wild-type lymphocytes into 24 clusters including 19 *cd79a*-expressing B-lineage clusters (19804 cells, 86.4%), 2 *lck*-expressing T/NK cell clusters (1017 cells, 4.4%), and 3 *gata1*-expressing erythrocyte clusters, which seemed to be contaminated by FSC^low^ erythrocytes during cell sorting (**Figure 5A**). Excluding these T/NK and erythroid clusters from wild-type and the corresponding *rag1* mutant cells, PCA analysis of 19804 B-lineage cluster cells from three wild-type and 12989 cells from *rag1* mutant cells based on the proportion of cells in each cluster was performed, confirming genotype-dependent separation (**Supplementary Figure S5B**).

Next, 19804 wild-type cells in B-lineage clusters were separated into 19 clusters and annotated according to subsequent analyses of gene expression patterns and *rag1*-dependency as described below (**Figure 5B**). A heat map of gene expression levels was constructed using each cluster-specific representative genes (p<0.05) and genes known to be involved in B-cell development in mammals and zebrafish (**Figure 5C**; **Supplementary Tables S2**, **S5**). *cd79a* and *pax5* were constitutively expressed in all the B-lineage clusters. Indication of wild-type B-lineage clusters on t-SNE visualization of B-lineage cells of both wild-type (19804 cells) and *rag1* mutant (12989 cells) identified *rag1*-independent clusters (shared by wild-type and *rag1* mutant) of early B cell development and *rag1*-dependent clusters (absent in *rag1* mutant) of late B cell development requiring V(D)J recombination of immunoglobulin genes (**Figure 5D**). In contrast, re-clustering of B-lineage cells from the mixture of wild-type and *rag1* mutants did not generate any *rag1* mutant-specific clusters (data not shown).

Three clusters with high *rag1* expression [cycling *rag1* ^high^ B, *rag1* ^high^ B (1–2)] and two clusters with low *rag1* expression [*rag1* ^low^ B (1–2)] were identified (**Figures 5C, E**). Whereas *the rag2* expression pattern basically coincided with *rag1*, expression of *DNA nucleotidylexotransferase* (*dntt*) and *sid1*, an ortholog of zebrafish *sid1* that is considered to be orthologous to mammalian *VPREB1* (60), was observed only in the clusters with high *rag1* expression (**Figures 5C, E**). In *the rag1* mutant, cells merging with these *rag1*-expressing wild-type clusters were highly accumulated (**Figure 5D**), suggesting developmental arrest at this stage. Accumulation of *the rag1*:EGFP^high^ population in *the rag1* mutant in the FACS analysis (**Figures 2B, C**) supports this notion.

Six Clusters [common lymphoid progenitors (CLP) (1–2), cycling *rag1* ^–^ B (1–4)] were commonly present in the wild-type and *rag1* mutants and did not express *rag1* (**Figures 5C–E**). Among these clusters, genes labeling proliferating cells (*mcm5*, *pcna*, *dut*, *rrm2, and mki67*) were commonly expressed in 4 cycling *rag1* ^–^ B clusters (**Figures 5C, E**), suggesting that these clusters represent the stage of cell proliferation before heavy chain gene rearrangement. *csf1rb* marking hematopoietic stem cells (27) was expressed in CLP (1-2) clusters, and *cebpa* marking hematopoietic stem cells as well as myeloid lineages (27) was expressed in part of the CLP (1) cluster (**Figures 5C, E**), suggesting that these cell populations are the earliest progenitors of the sorted lymphocytes.

Eight clusters [*mpeg1* ^+^ B (1–8)] were *rag1*-dependent without *rag1* expression (**Figures 5C–E**), suggesting that they were the most mature B-cell populations after completion of immunoglobulin gene rearrangement. In contrast to the expression of *ebf1*, *myb*, and *si-dkey261h17.1* in early B cell-populations, these late clusters commonly express *cd22l*, *dusp2*, and *mpeg1* (**Figures 5C, E**; **Supplementary Figure S7**). *ebf1*-expressing immature B cells, but not *cd22l*-high, relatively mature B cells, were present in *the rag1* mutant, reflecting the qPCR results (**Figure 2D**).

Unlike zebrafish, in which clusters expressing *igD* and teleost-specific *igT* were separated (27, 61), *igT* has not been identified in medaka (62), and *immunoglobulin m heavy chain* (*ighm)*-expressing cells were scattered (**Supplementary Figure S7**). In zebrafish, several genes including *ccr2* and several genes including *irf8* were co-expressed with *igcz* (*igT*) and *igcd* (*igD*), respectively (27). However, cells expressing one of the *ccr2* paralogs and *irf8* did not form independent clusters in medaka (**Supplementary Figure S7**), indicating no sign of separation of the relatively mature B-cell populations.

### 3.6 Identification of T and NK cells in the kidney

**Figure 6:**
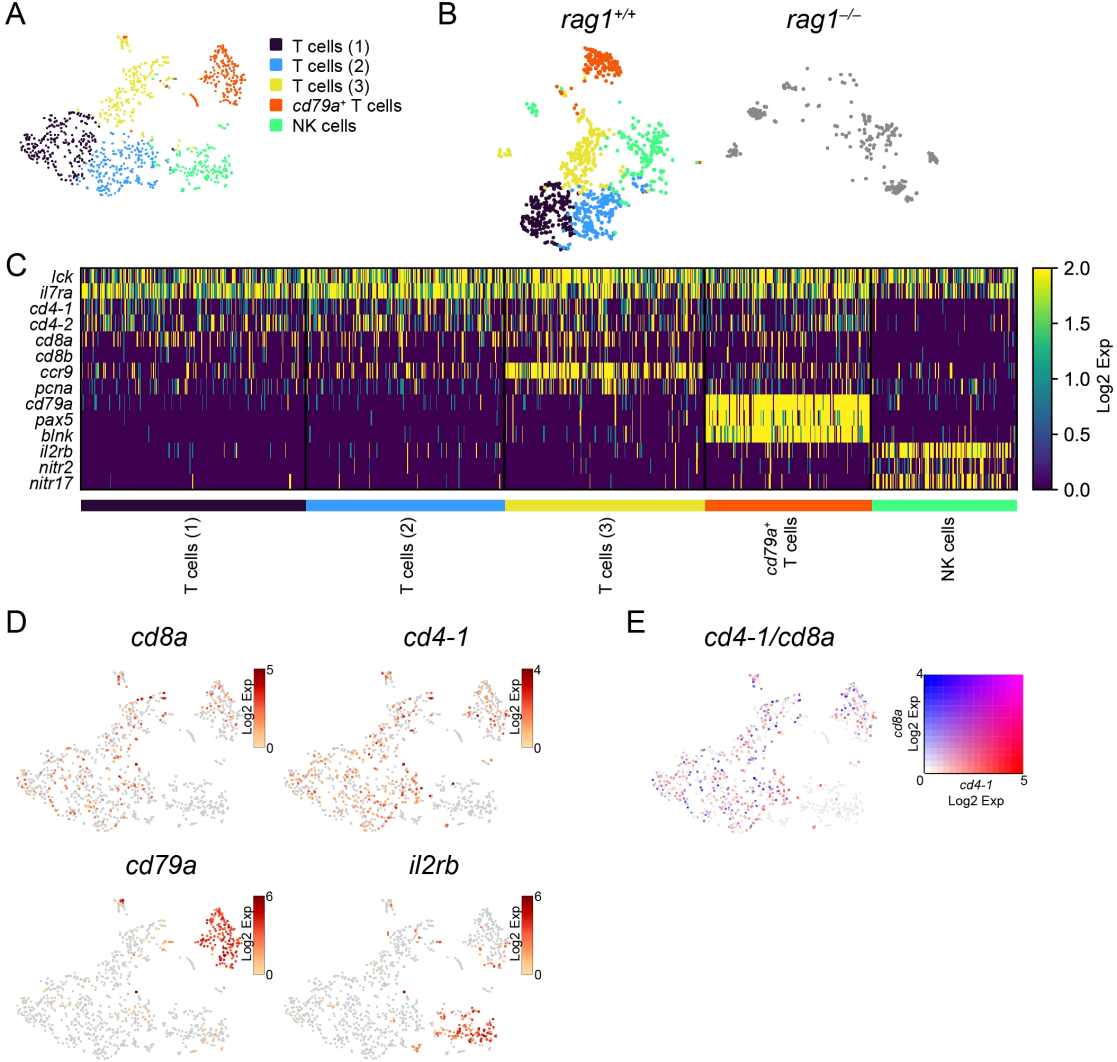
Categorization of T/NK cells in the kidney. **(A)** t-SNE visualization of 1037 T/NK cells from wild-type kidney marrow. Clusters with annotations are also presented. **(B)** t-SNE visualization of T/NK cells from the wild-type (left) and *rag1* mutant (right) kidney marrow. Wild-type cells are colored based on the clusters in **(A)**. Mutant cells are shown in gray. **(C)** Heatmap of marker gene expression in each T/NK-cell cluster in the wild-type. **(D)** t-SNE visualization of T/NK cells from wild-type kidney marrow. Expression levels of indicated genes are shown. **(E)** t-SNE visualization of T/NK-lineage cells from wild-type kidney. Merged expression levels of *cd4-1* and *cd8a* are shown.

To characterize T and NK cells in the kidney, *lck*-expressing T/NK clusters in wild-type kidney lymphocytes (**Figure 5A**) were focused and re-clustered. To this end, 1037 cells were separated into five clusters, all of which expressed *lck* and *il7ra* (**Figure 6A**; **Supplementary Table S6**). Indication of these wild-type T/NK-lineage clusters on t-SNE visualization of T/NK cells of both wild-type (1037 cells) and *rag1* mutant (276 cells) identified one *rag1*-independent (NK cells) and four *rag1*-dependent clusters (T cells (1–3), *cd79a^+^* T cells) (**Figure 6B**). The heatmap and t-SNE profiles of these clusters showed that *rag1*-dependent clusters commonly contained cells expressing *cd4* paralogs (*cd4-1* and *cd4-2*) or *cd8* (*cd8a* and *cd8b*) (**Figures 6C, D**; **Supplementary Figure S8**), suggesting that they are T cells. Based on the expression levels at the individual cell level, *cd4-1* and *cd8a* were separately expressed in the cells of T-cell clusters (**Figure 6E**), in contrast to overlapping expression in thymic DP clusters (**Figure 3F**), suggesting that they are mature CD4^+^ or CD8^+^ T cells.

One of these T-cell clusters (*cd79a^+^* T cells) also expressed B-cell markers *cd79a*, *pax5*, and *blnk* (**Figures 6C, D**). Among the three *cd79a^–^* T-cell clusters [T cells (1–3)], the T cells (3) cluster showed higher expression of genes including *ccr9* and *pcna* (**Figure 6C**; **Supplementary Table S6**).

The *rag1*-independent cluster (NK cells) expressed *il2rb*, *nitr2*, and *nitr17* (**Figures 6C, D**; **Supplementary Figure S8**), whose zebrafish orthologs are known markers of NK cells (25, 26, 59), suggesting that this population is composed of NK cells.

### 3.7 Identification of lymphoid progenitor cells already committed to the T lineage in the kidney

**Figure 7:**
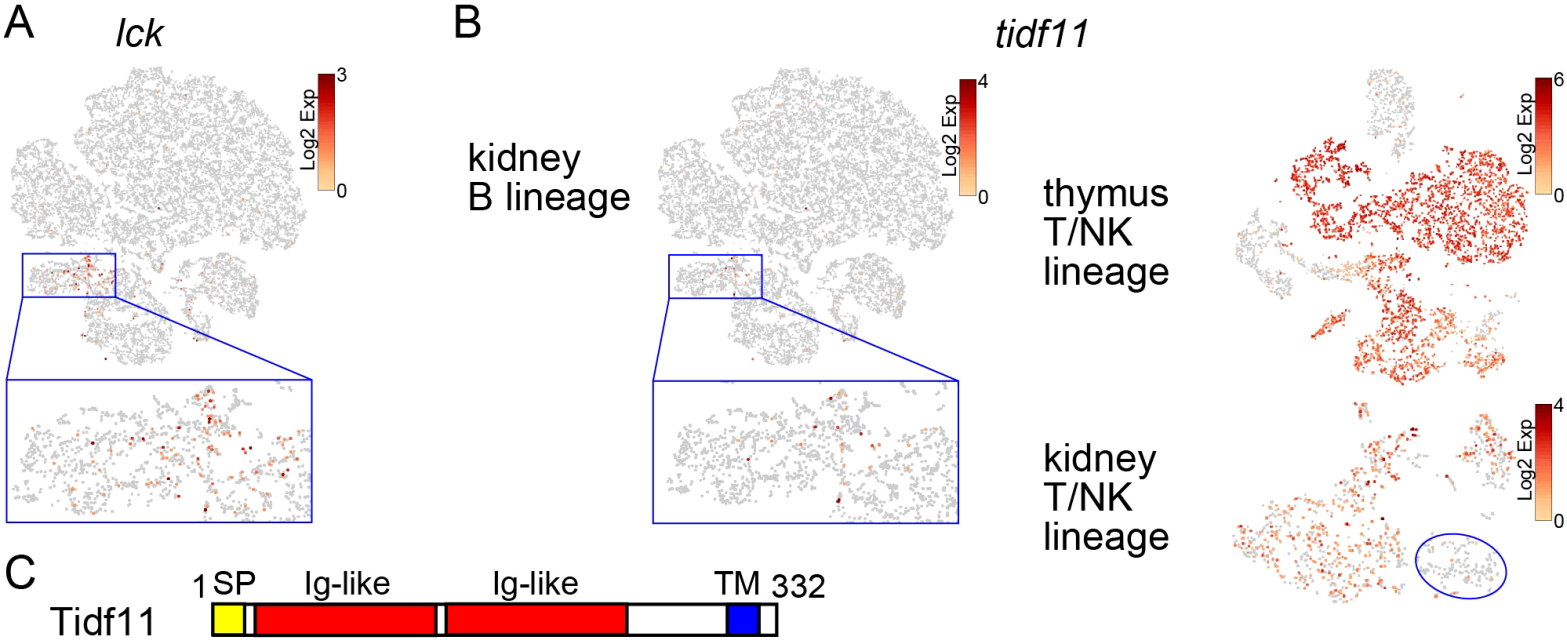
Identification of T-cell precursors in the kidney. **(A)** t-SNE visualization of B-lineage cells from the wild-type kidney marrow. Expression level of *lck* is shown. A magnified image of the blue square area is also shown. **(B)** t-SNE visualization of B-lineage cells from wild-type kidney marrow, T/NK-lineage cells from wild-type thymus, and T/NK-lineage cells from wild-type kidney. Expression level of *tidf11* is shown. A magnified image of the blue square area is also shown. The NK cluster of the kidney T/NK-lineage cells is indicated by a blue circle. Note that there are few cells expressing *tidf11* in the NK cluster. **(C)** Domain structures of Tidf11 protein with 332 amino acids. Signal peptide (SP), two immunoglobulin-like (Ig-like) domains, and transmembrane domain (TM) are indicated in yellow, red, and blue, respectively.

Another T lineage was identified in *cd79a*-expressing B-lineage cell clusters in the wild-type (**Figure 5A**). Cells expressing the T/NK-lineage markers *lck* and *il7ra*, but not *cd4*/*cd8*, were present in a portion of the CLP cluster and merged with *cebpa*-expressing cells (**Figure 7A**; **Supplementary Figure S9A**; **Figure 5E**). These cells also expressed *notch1a*, one of the orthologs of mammalian *notch1* whose signaling is involved in T-cell fate decision (63, 64) (**Supplementary Figure S9A**).

Some genes including *interferon-induced transmembrane protein 5* (*ifitm5*) and *t-cell-specific immunoglobulin domain family 11* (*tidf11*), were specifically expressed in the identical population in kidney and T cells, except for DP clusters in the thymus (**Figure 7B**; **Supplementary Figure S9B**). In the kidney, they were also expressed in mature T cells but not in NK cells (**Figure 7B**; **Supplementary Figure S9B**). *ifitm5* is one of the orthologs of mammalian *ifitm* family genes that encode transmembrane proteins involved in helper T-cell differentiation and immune response against cancer and viral infection (65, 66). *tidf11* is one of 18 family member genes on chromosome 17 (**Supplementary Table S7**). At least five other family member genes (*tidf3*, *tidf7*, *tidf10*, *tidf12*, and *tidf16*), for which the Ensembl gene ID was assigned, showed similar expression patterns to *tidf11* (data not shown). As *tidf* family genes, except three members (*tidf8*, *tidf9*, *tidf13*) whose start codons have not been identified in this study, are predicted to encode transmembrane proteins with two immunoglobulin domains (**Figure 7C**), this family has been named the *t-cell-specific immunoglobulin domain family*. These results suggest that this CLP cluster contains cells committed to the T lineage and identified *ifitm5* and *tidf* family genes already expressed at the T-lineage commitment stage and also in mature T cells.

## 4 Discussion

In this study, we characterized T-cell development in the thymus and B-cell development in the kidney of medaka using scRNA-seq analysis of lymphocytes in the wild-type and *rag1* mutants. As a result, we found that DP cells, not SP cells, are the most developed T cells in the thymus, and lymphoid progenitor cells are already committed to the T lineage in the kidney. Thus, we clarified difference between medaka and zebrafish in molecular mechanisms underlying lymphocyte development.

Although the zebrafish *rag1* mutant was established and shown to have problems in adaptive immune response due to the lack of mature lymphocytes (17, 67, 68), the compatible immunodeficient mutant of the other established teleost model is useful in terms of comparative immunity for understanding variations in fish immune systems. Accordingly, we established a *rag1* mutant of medaka and characterized its phenotype. The mutant showed defects in the VDJ recombination of *igh* and *tcrb* (**Figure 1**) and exhibited B-cell development in the kidney and T-cell development in the thymus (**Figures 1**, **2**). Regarding thymic T cells, although no obvious reduction in the area of *lck*:EGFP^+^ thymocytes was observed in the *rag1* mutant, reduction in *lck*:EGFP intensity suggests a reduction in *lck*:EGFP^+^ cells in the larval *rag1* mutant thymus, and a reduction in the area of the largest portions of thymic transverse sections suggests a reduction in the thickness of the adult *rag1* mutant thymus (**Figure 1**). These phenotypes are similar to those of mammalian and zebrafish *rag1* mutants (7, 17, 67, 68), suggesting the conservation of *rag1* function in jawed vertebrates (**Table 1**).

**Table 1:** Comparison of lymphopoiesis among mouse, zebrafish, and medaka.

Feature	Mouse	Zebrafish	Medaka
*rag1*-dependency	Necessary for B- and T- cell development (7).	Necessary for B- and T- cell development (67, 68).	Necessary for B- and T- cell development (this paper).
B-cell lineage commitment marker	*egf1* (44)	*ebf3a* (27)	*ebf1* (this paper)
DP and SP T cells in the thymus	Close to 90% of thymocytes are DP T cells. Approximately 10% of thymocytes are CD4 or CD8 SP T cells (69).	Majority of the thymocytes express *cd4* or *cd8*. Among them, *cd4* or *cd8* SP T cells are more than DP T cells (27).	70% of thymocytes are DN T cells. No clear *cd4* or *cd8* SP T-cell populations. All DP cells retain *rag1* expression (this paper).
T cell precursors in bone marrow/ kidney	Tentative expression of PIRs in pre-thymic progenitors in mouse (70). Loss of the other lineage potential of thymocytes is completed by double-negative 3 stage (71).	HSPCs share expression of *cebpa*, *csf1rb*, and *fli1* with ETPs (27).	T-lineage commitment (expression of *lck*, *il7ra*, *tidf* family) in a part of CLPs (this paper).

DN, double-negative; DP, double-positive; SP, single-positive; HSPCs, hematopoietic stem and progenitor cells; ETPs, early T-cell progenitors; CLPs, common lymphoid progenitors.

For a detailed characterization of T- and B- cell development, we performed scRNA-seq analysis of lymphocytes in the thymus and kidney. In addition to the analysis of wild-type cells, analysis of *rag1* mutant cells enabled us to distinguish early lymphocyte progenitors independent of Rag1 function and relatively mature populations requiring V(D)J recombination of *immunoglobulin* and *tcr* genes.

After immigration of ETPs from hematopoietic organs, αβ T-cell development follows the stages from CD4^–^CD8^–^ DN, CD4^+^CD8^+^ DP, to CD4^+^CD8^–^ or CD4^–^CD8^+^ SP, and the majority of the developing T cells in the thymus is DP in mammals (**Table 1**) (8, 69). In zebrafish, scRNA-seq analysis revealed that the majority of T-lineage cells express *cd4-1* or *cd8a*, among which *cd4* SP or *cd8* SP cells are more abundant than DP cells (**Table 1**) (https://dr-marrow-thymus.cells.ucsc.edu) (27). Furthermore, FACS analysis of ginbuna crucian carp thymocytes using CD4-1 and CD8a antibodies showed the existence of DP (26%), CD4 SP (20%), and CD8 SP (22%) populations (72). However, in our medaka scRNA-seq analysis of thymic lymphocytes, as many as approximately 70% of the T/NK-lineage cells were DN (**Figure 3**). It should be noted that *rag1* is already expressed in ETPs and its expression is maintained through αβ T-cell development, and developmental arrest in the *rag1* mutant starts at the ETP stage (**Figure 3**). These results suggest that Rag1 function for *tcrb* gene rearrangement has already begun shortly after thymic immigration of T progenitors.

In addition to the unexpectedly low proportion of the DP population, it is surprising that neither *cd4^+^cd8* ^–^ nor *cd4^–^cd8^+^* clusters were present in the medaka thymus (**Figure 3**). At the individual cell level, following the preceding expression of *cd4-1* in the cycling T (3) cluster, cells in thymic DP T clusters exhibited overlapping expression of *cd4-1* and *cd8a* (**Figure 3**). This is in contrast to kidney T cells, which are considered mature repopulated T cells, with separated expression of *cd4-1* and *cd8a* (**Figure 6**). Defining the cells expressing *cd4-1* or *cd4-2* as *cd4^+^* cells, and those expressing *cd8a* or *cd8b* as *cd8^+^* cells, the proportions of *cd4^+^cd8^+^* DP, *cd4^+^cd8^–^* SP, *cd4^–^cd8^+^* SP, and *cd4^–^cd8^–^* DN cells in the thymic DP cluster cells were 85.8 ± 0.3%, 1.7 ± 0.7%, 9.0 ± 0.5%, and 3.4 ± 0.5%, respectively, whereas in the kidney T-cell clusters they were 1.8 ± 1.2%, 47.0 ± 2.6%, 18.6 ± 1.2%, and 32.6 ± 3.6%, respectively. Although there were a small number of SP cells in the thymic DP clusters, these SP cells did not have any differentially expressed genes compared with DP cells (data not shown), suggesting that these rare SP cells are not discrete populations with dynamic transcriptomic changes. In addition, in contrast to zebrafish, in which all SP cells and some DP cells ceased *rag1* expression (27), medaka DP clusters in our study kept *rag1* expression (**Figure 3**). These observations imply that thymic DP cells emigrate from thymus just after *tcra* rearrangement and positive selection before ceasing expression of *rag1* and becoming SP cells (**Table 1**). Analysis of protein expression on the cell surface using antibodies against medaka Cd4 and Cd8 will further assess findings in this study.

In the thymus, two separate DP clusters were identified (**Figures 3**, **4**). GO analysis of differentially expressed genes between these clusters indicated that cells in the DP T (1) cluster had higher expression of the “DNA replication-dependent chromatin assembly” and “spindle elongation” pathway genes, indicating that they are more proliferative. In contrast, cells in DP T (2) cluster have higher expression of “T cell differentiation” and “T cell activation” pathway genes including *cd4*, *cd8*, and *ccr9*. *tespa1* required for positive selection is also highly expressed in the DP T (2) cluster (**Figure 4**), raising the possibility that the DP T (1) cluster includes proliferating cells before *tcra* gene rearrangement and the DP T (2) cluster includes cells after *tcra* gene rearrangement undergoing positive selection.

According to the continuous expression of *rag1*, the gene rearrangement of *tcrb* and *tcra* seems to occur serially (**Figure 3**). In this study, the timing of the gene rearrangement of *tcrb* and *tcra* was not studied. The gene encoding *pre T-cell antigen receptor alpha* (*PTCRA*), constituting pre T-cell receptor together with TCRβ (73) for β selection, has not been identified in teleosts. Hence, the timing of β selection and positive/negative selection of developing T cells was not completely identified in this study.

As non-αβ T lineages, three cell types were identified according to their marker gene expression patterns, although these cell types were not distinguished as separate clusters (**Figure 4**). In addition to *trdc*, expression of *sox13* and *blk*, which are specifically expressed in γδ T cells in zebrafish (27), defined medaka γδ T cells (**Figure 4**). *lag-3*, an ortholog of the gene encoding an inhibitory receptor related to CD4 (56), was expressed in γδ T cells (**Figure 4**). This fits the case of mice, where the proportion of *lag-3*-expressing cells is much higher in γδ T cells than in αβ T cells (47). *zbtb16a*, an ortholog of the marker gene of iNKT cells (57, 58), was highly expressed in cell masses other than γδ T cells (**Figure 4**). However, it is unclear whether these cells have invariant TCR chains, as seen in mammals (74).

The other cell population is independent of *rag1* and expresses orthologs of the zebrafish NK cell markers, *il2rb* and *nitr2* (25, 26, 48) (**Figures 3**, **4**). Although it is possible that related innate lymphoid cells (ILCs) are also included in this cell population, this study did not analyze enough cells to separate them, and defined this population as NK cells.

During B-cell development in the kidney, *rag1* was expressed in one wave, suggesting that gene rearrangement of *igh* and *igl* seems to occur serially. The clusters expressing *rag2*, which encodes the partner protein of Rag1 for antigen receptor rearrangement, were identical to those expressing *rag1*. However, *rag1* ^high^ B and *rag1* ^low^ B clusters showed comparable expression levels of *rag2* (**Figure 5**). This difference in the timing of expression of these genes were also observed in zebrafish (27). The expression of an ortholog of zebrafish *sid1*, which is considered to be orthologous to mammalian *VPREB1* in *rag1* ^high^ clusters expressing *dntt*, implies that these clusters include the pre-B cell stage expressing rearranged *igh*, together with *VpreB* (**Figure 5**). However, the timing of the gene rearrangement of *igh* and *igl* was not studied in this study. The expression of *ighm* does not reflect the expression of rearranged *igm* because germline transcripts before rearrangement were also included, despite the fact that cells with less than 25 reads were judged as negative in **Supplementary Figure S6**. It is to be noted that although *ebf3a* is considered as a B-lineage commitment factor in zebrafish (27), our expression analysis suggests that *ebf1* has such function in medaka, as in mammals (44) (**Figure 5**, **Table 1**).

In zebrafish, the most developed B cells in the kidney were clearly separated into IgD and IgT B cells according to the expression of *ig* and accompanying genes, including *irf8* (igD B) and *ccr2* (igT B) (27). In contrast, *igT* has not yet been identified in medaka (62). Indeed, cells expressing *ighm*, *irf8*, and *ccr2* were evenly distributed in the eight most mature *mpeg1* ^+^ B clusters (**Figure 5**; **Supplementary Figure S7**), indicating no sign of separation of the most mature B cells. *mpeg1* was reported to be expressed by a subpopulation of B cells as well as macrophages (75). This was also true in medaka; among the B-lineage cells, *mpeg1* was expressed by the most developed B cells (**Figure 5**).

We found *rag1*-independent NK clusters and *rag1*-dependent T clusters in *lck*-expressing T/NK clusters in the kidney (**Figure 6**). As mentioned above, T cells expressing *cd4* and *cd8* were not separated into independent clusters, possibly because they were not activated. The only medaka orthologue of *foxp3* (ENSORLG00000025857) found to date, a marker of CD4^+^ regulatory T cells (Tregs) (76), was widely expressed in both *cd4^+^* and *cd8* ^+^ T cells (data not shown). Notably, there was a cluster of cells expressing both T- and B- cell markers (**Figure 6**). The function of this possibly novel cell population with a unique expression pattern is of particular interest.

A recent study identified zebrafish ETPs, which shared the expression of genes including *cebpa*, *csf1rb*, and *fli1* with HSPCs in the kidney (27). However, none of these genes were commonly expressed in ETPs in the thymus and CLPs in the kidney in our medaka study, suggesting that timing of ETP immigration or their gene expression patterns are not conserved between zebrafish and medaka (**Table 1**). Indeed, cells expressing *lck* and *il7ra* were present in part of the CLP cluster in the kidney (**Figure 7**), suggesting that they are already committed to the T lineage. The specific expression of *notch1a* (**Figure 7**), one of the orthologs of mammalian *notch1* involved in T-lineage commitment from T/B progenitors (63, 64), supports this notion. As the lymphocyte gate used for cell sorting of thymocytes covered the gross *lck*:EGFP^+^ cells (**Supplementary Figure S4**), it is unlikely that ETPs were omitted from our scRNA-seq analysis. In mice, Paired immunoglobulin-like receptors (PIRs) are expressed by prethymic T progenitors (**Table 1**) (70). However, the expression of PIRs in thymocytes is limited to the earliest intrathymic stage (70), and the other lineage potential of thymocytes is retained until the DN3 stage (71). In contrast, despite sharing the immunoglobulin domain with PIR, medaka Tidf family proteins are continuously expressed from the T-cell commitment stage in the kidney to mature T cells (**Figure 7**). Orthologs of *tidf* family genes are found in many species in the superorder *Acanthopterygii* including medaka and tetraodon, but not in other teleosts including zebrafish and salmon, according to the Ensembl database (data not shown), possibly reflecting the diversity of molecular mechanisms of T-lineage commitment in teleosts.

In summary, our study has characterized cellular and molecular basis of T- and B- cell development. In comparison with zebrafish mechanisms, we have shown unique features of medaka lymphopoiesis including the absence of the SP T-cell population in the thymus, and the presence of lymphoid progenitor cells expressing novel *tidf* family genes already committed to T lineage in the kidney. We believe that this information, in combination with future studies on immune function, will contribute to comparative immunity in teleosts in the context of evolutionary adaptation to the environment.

## Data Availability

The datasets presented in this study can be found in online repositories. The names of the repository/repositories and accession number(s) can be found in the article/**Supplementary Material**.
