# Supplementary material for: Single-cell transcriptome analysis of medaka lymphocytes reveals absence of fully mature T cells in the thymus and the T-lineage commitment in the kidney
Source: Front Immunol. 2025 Jan 10;15:1517467. doi: 10.3389/fimmu.2024.1517467 (PMC11759298; doi:10.3389/fimmu.2024.1517467)
Supplement: Supplementary file 2 [file DataSheet2.pdf]

**Supplementary Material**

## 1.1 Supplementary Information

### *lck:egfp* transgenic medaka

To visualize T cells and NK cells, *lck:egfp* transgenic medaka, which express EGFP under the control of *the lck* promoter, have been established. In this strain, EGFP expression in developing T cells in the thymus was first detectable at stage 31 (**Figure S3**) and then EGFP$^{+}$ cells accumulated in the thymus, similar to the previously reported *lck:egfp* line (Bajoghli et al., 2015). In adult fish, EGFP-expressing T cells were densely populated in the thymus and also detected in the gills and intestine (**Figure S3**). Flow cytometric analysis of whole kidney marrow cells identified an EGFP$^{+}$ cell population within a population with a lymphocytic light scatter profile. We performed qPCR analysis of fluorescence-activated cell sorting (FACS)-sorted EGFP$^{-}$ and EGFP$^{+}$ lymphocytes. In addition to *lck*, T-cell markers *cd8b* and *cd4-1*, and the NK-cell marker *nitr17* were specifically expressed in EGFP$^{+}$ cells. In contrast, the B-cell marker *cd79a* was specifically expressed in EGFP$^{-}$ cells. These results suggested that *lck*:EGFP expression labels T and NK cells.

**1.2 Supplementary Figures**

# Figure S1

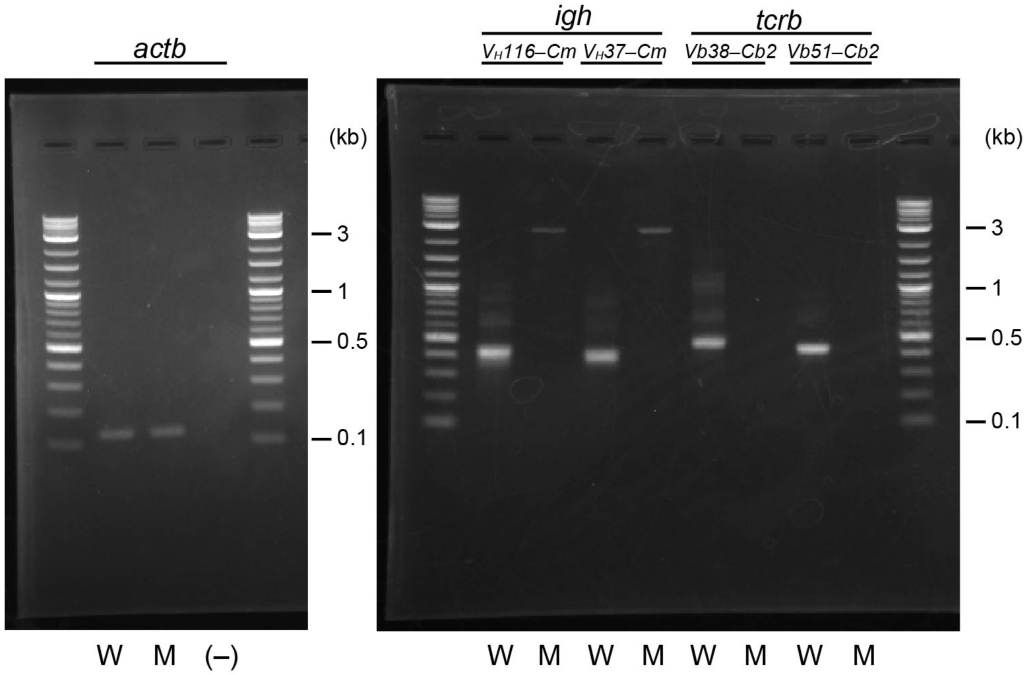


**Figure S1 Defective rearrangements of antigen receptor genes in *rag1* mutants.**

Reverse transcription PCR (RT-PCR) of whole kidney cells of the indicated genotypes using the indicated primers to detect VDJ-recombined *igm* and *tcrb*. *actb* expression was used as a standard. (−), water control; W, wild-type; M, $rag1^{-/-}$. The 3kb bands of *igh* in $rag1^{-/-}$ are non-specific amplicons. The entire original gel images of Figure 1C.

Figure S2

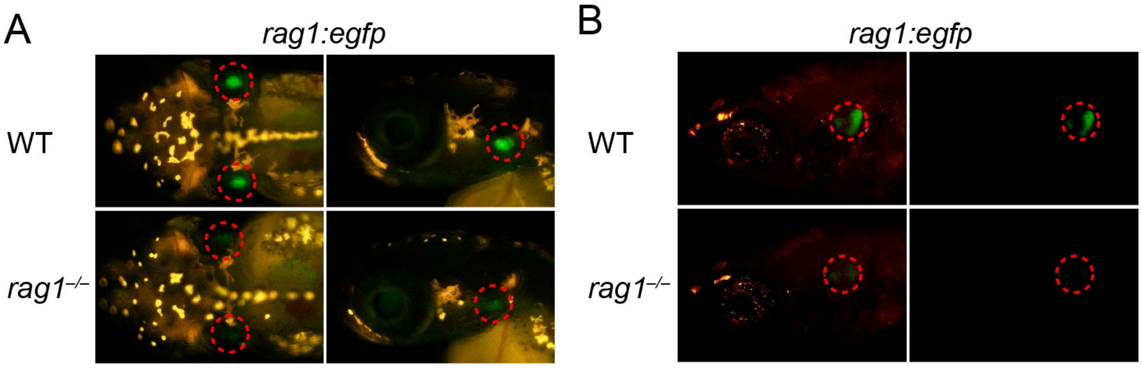


**Figure S2** ***rag1*** **mutation affects developing T cells.**

**(A)** *rag1:*EGFP expression in larval thymocytes at 10 days post-fertilization (dpf). Dorsal (left) and lateral (right) views of the indicated genotypes. Dotted circles indicate the thymus. **(B)** *rag1:*EGFP expression in adult thymocytes at 2 months post-fertilization (mpf). Lateral views of the indicated genotypes with (left) and without (right) visible-light transmission. Dotted circles indicate the thymus. Note the reduced EGFP-positive thymocytes in the *rag1* mutants.

Figure S3

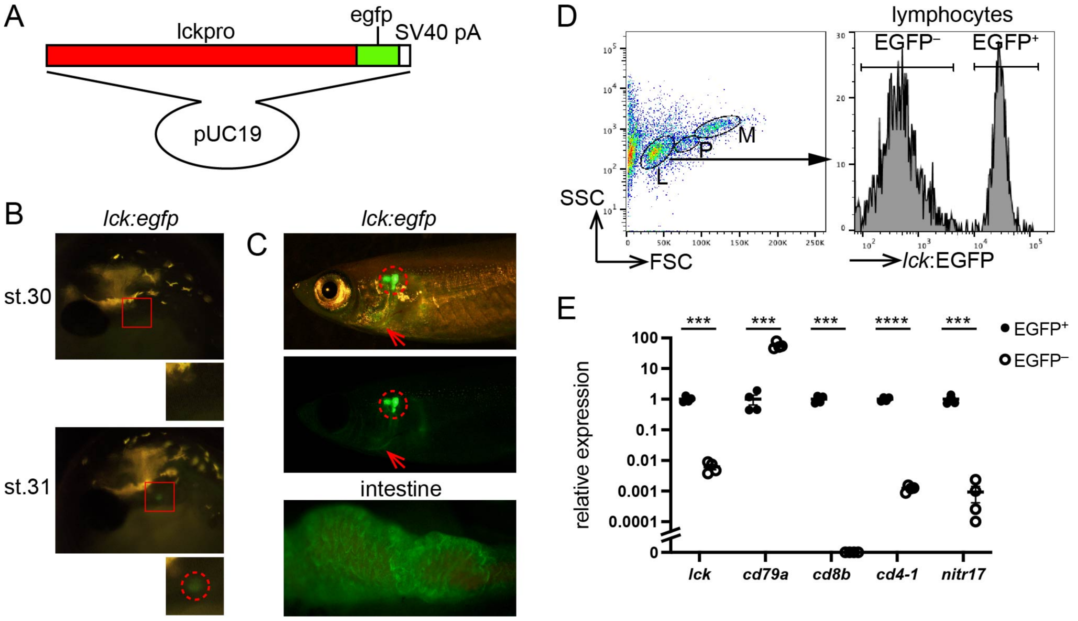


**Figure S3 *lck:egfp* transgenic medaka for labeling T and NK cells.**

**(A)** Construct for *lck:egfp* transgenesis. The 4.7kb of the medaka *lck* promoter, *egfp*, and SV40 polyA, were cloned into the linearized pUC19 plasmid. **(B)** *lck:*EGFP signals in the larval thymus. Lateral views. Magnified images of red square areas are also shown. Dotted circles indicate the thymus. Note that *lck:*EGFP signals were not detected at stage 30 (top), but at stage 31 (bottom) in the thymus. **(C)** *lck:*EGFP signals in adult fish. Lateral views with (top) and without (middle) visible-light transmission and enlarged images of the intestine with a higher laser intensity (bottom). Note intense EGFP expression in the thymus (dotted circles), gills (arrows), and intestine. **(D)** Flow cytometric profiles of adult whole kidney marrow (WKM) cells with *lck:egfp* transgene. Light scatter profiles (left). Circles indicate lymphocyte (L), precursor (P), and myelomonocyte (M) populations. *lck:*EGFP signal levels in lymphocytes (right). The $EGFP^+$ and $EGFP^-$ populations were sorted for qPCR analysis. FSC, forward scatter; SSC, side scatter. **(E)** Quantitative PCR (qPCR) analysis of $EGFP^+$ and $EGFP^-$ lymphocytes in the kidney (n = 4). The average expression level in $EGFP^+$ cells is normalized to 1. Data represent the mean ± standard error of the mean (SEM), and statistical significance was determined using an unpaired two-tailed t-test. ***, $p < 0.001$; ****, $p < 0.0001$. The results represent one of the two independent experiments with similar results.

Figure S4

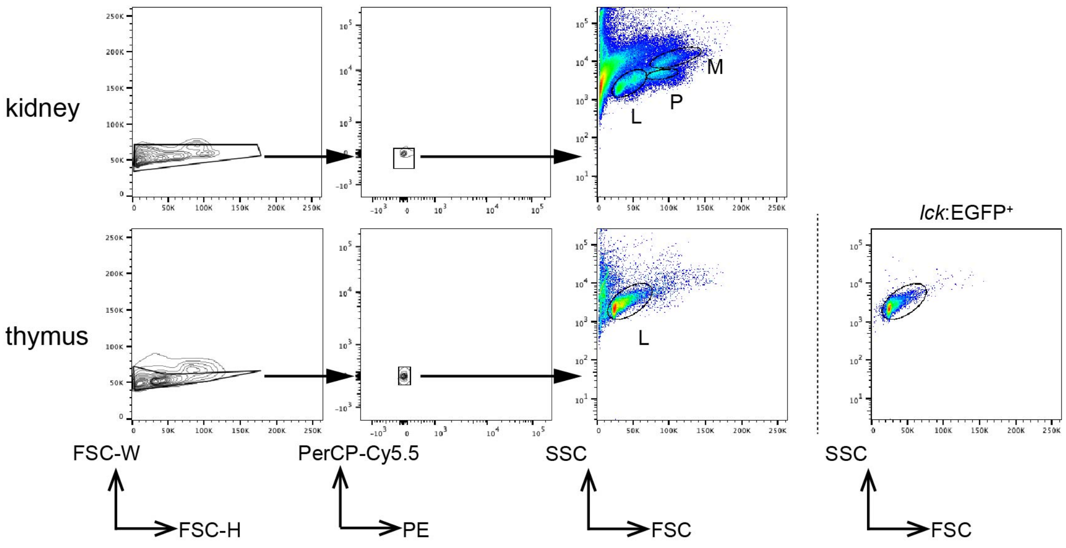


**Figure S4 Sorting strategy of kidney and thymus lymphocytes for scRNA-seq.**

Using flow cytometry, single-cell suspensions of the kidney (top) and thymus (bottom) were first gated to exclude doublet cells (left), followed by gating to exclude dying cells with autofluorescence (middle). The resulting cells were gated by light scatters to sort the lymphocytes (right). The light scatter profile of the *lck*:EGFP$^+$ cells in the thymus is also shown on the far right. L, lymphocytes; P, precursors; M, myelomonocytes; FSC-H, forward scatter height; FSC-W, forward scatter width; PE, phycoerythrin; PerCP-Cy5.5, peridinin chlorophyll protein-cyanine 5.5; FSC, forward scatter; SSC, side scatter.

# Figure S5

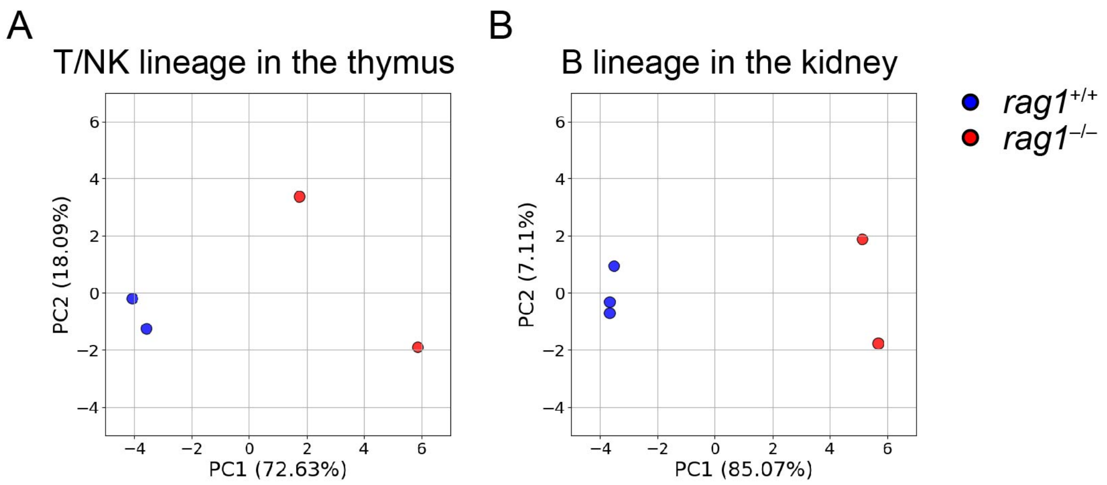


**Figure S5 Principal Component Analysis (PCA) indicating separation of cluster composition of wild-type and *rag1* mutants.**

PCA plots based on the cell proportions of each cluster for T/NK-lineage cells in the thymus (A) and B-lineage cells in the kidney (B).

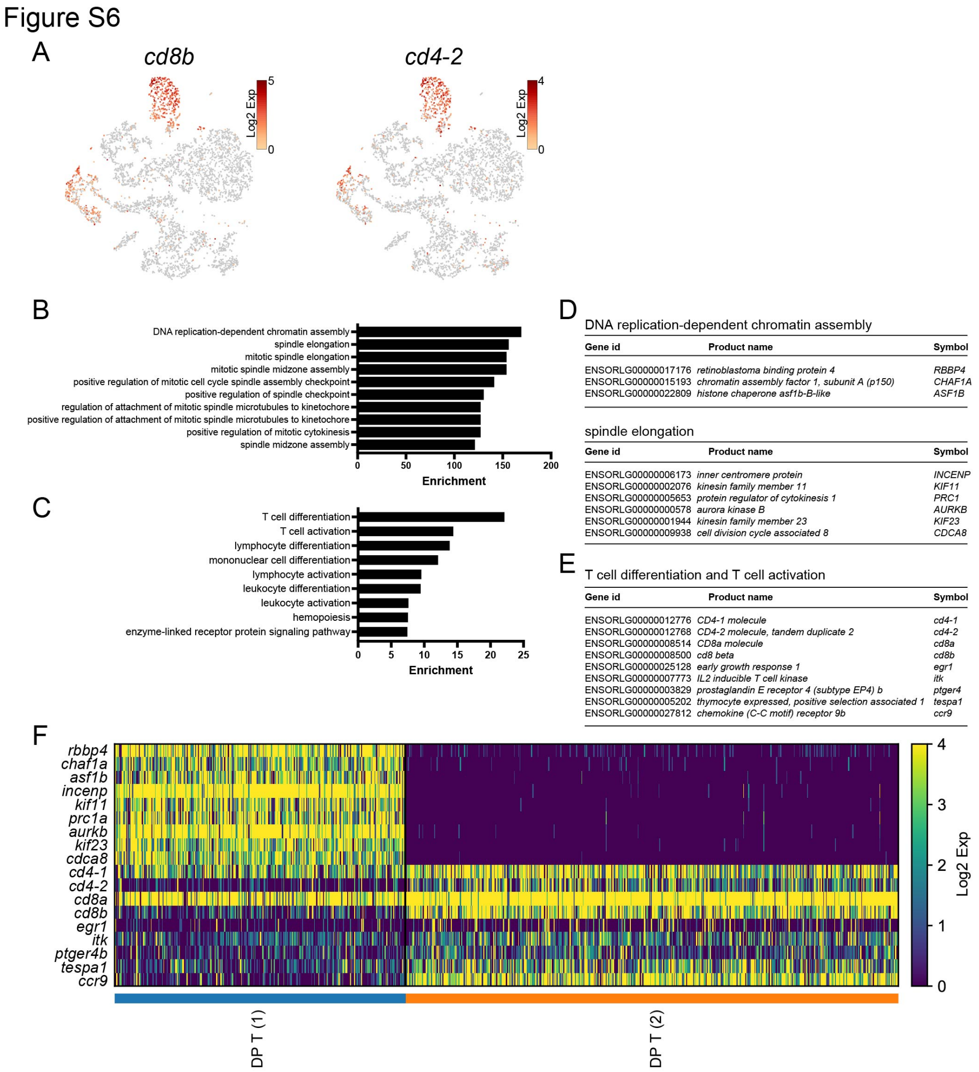


**Figure S6 Characterization of 2 double-positive (DP) clusters in the thymus.**

**(A)** t-SNE visualization of T/NK-lineage cells from the wild-type thymus. Expression levels of indicated genes are shown. **(B–C)** Pathways identified by GO enrichment analysis of differentially expressed genes between the two DP clusters. The top 10 out of 178 enriched pathways of the genes highly expressed in the DP T (1) cluster (B), and all nine enriched pathways of the genes highly expressed in the DP T (2) cluster (C) are shown. $\log_{10}q<0.05$. **(D)** List of "DNA replication-dependent chromatin assembly" (GO:0006335) and "spindle elongation" (GO:0051231) pathway genes in (B). **(E)** List of "T cell differentiation" (GO:0030217) and "T cell activation" (GO:0042110) pathway genes in (C). **(F)** Heat map of selected differentially expressed genes in each DP cluster in wild-type.

Figure S7

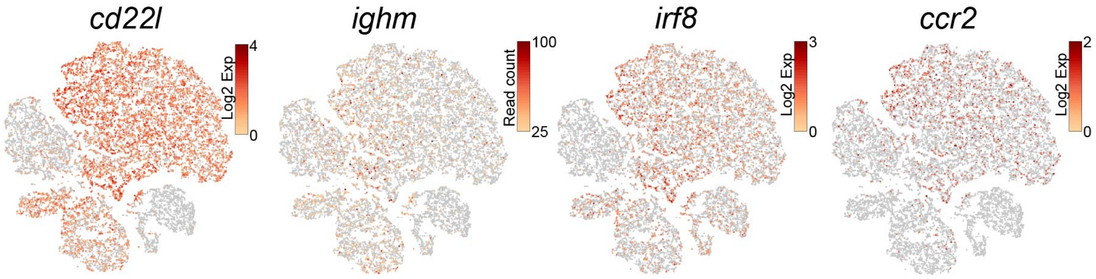


**Figure S7 No separation of mature B clusters in the kidney.**

t-SNE visualization of B-lineage cells from wild-type kidney marrow. Expression levels of the indicated genes are shown.

# Figure S8

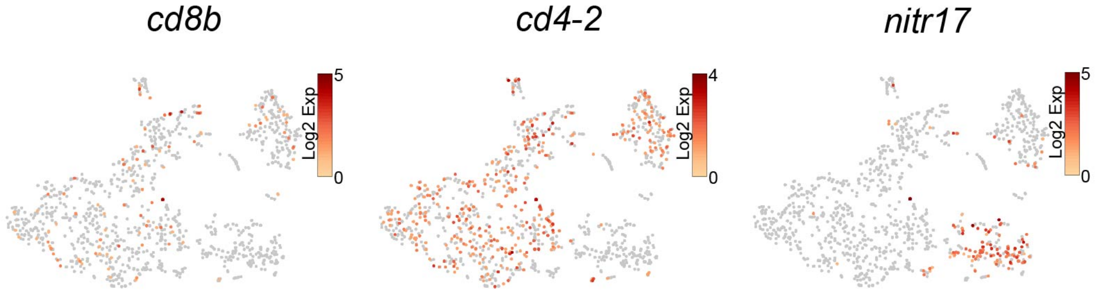


**Figure S8 T/NK cells in the kidney.**

t-SNE visualization of T/NK cells from wild-type kidney marrow. Expression levels of indicated genes are shown.

# Figure S9

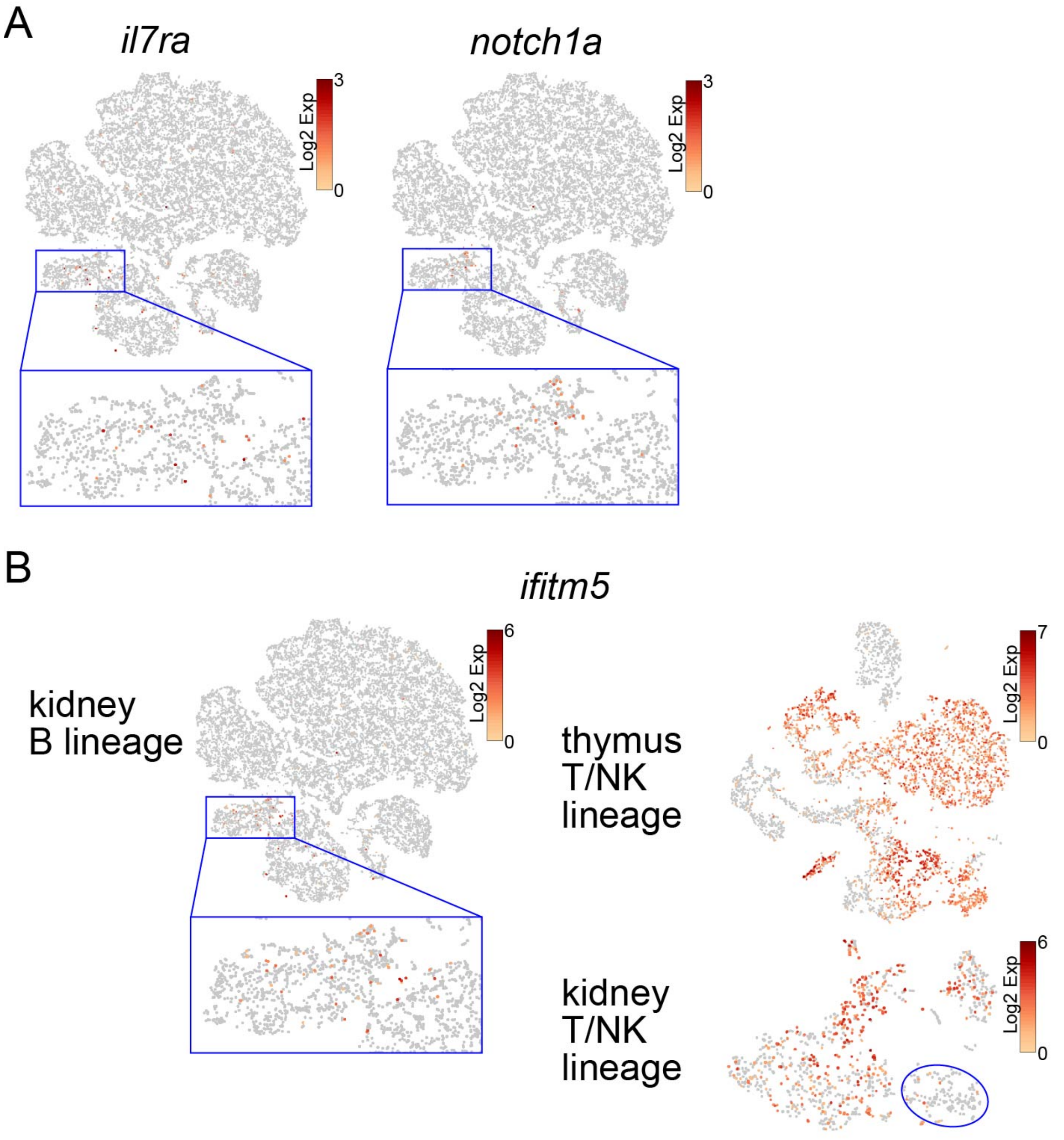


**Figure S9 T-cell precursors in the kidney.**

**(A)** t-SNE visualization of B-lineage cells from wild-type kidney marrow. Expression levels of the indicated genes are shown. Magnified images of the blue square areas are also shown. **(B)** t-SNE visualization of B-lineage cells from wild-type kidney marrow, T/NK-lineage cells from wild-type thymus, and T/NK-lineage cells from wild-type kidney marrow. The expression level of *ifitm5* is shown. A magnified image of the blue square area is also shown. The NK cluster of the kidney T/NK-lineage cells is indicated by a blue circle. Note that there are few cells expressing *ifitm5* in the NK cluster.
